# Optically-controlled long-term storage and release of thermal energy in phase-change materials

**DOI:** 10.1038/s41467-017-01608-y

**Published:** 2017-11-13

**Authors:** Grace G. D. Han, Huashan Li, Jeffrey C. Grossman

**Affiliations:** Department of Materials Science and Engineering, Massachusetts Institute of Technology, 77 Massachusetts Avenue, Cambridge, MA 02139 USA

## Abstract

Thermal energy storage offers enormous potential for a wide range of energy technologies. Phase-change materials offer state-of-the-art thermal storage due to high latent heat. However, spontaneous heat loss from thermally charged phase-change materials to cooler surroundings occurs due to the absence of a significant energy barrier for the liquid–solid transition. This prevents control over the thermal storage, and developing effective methods to address this problem has remained an elusive goal. Herein, we report a combination of photo-switching dopants and organic phase-change materials as a way to introduce an activation energy barrier for phase-change materials solidification and to conserve thermal energy in the materials, allowing them to be triggered optically to release their stored latent heat. This approach enables the retention of thermal energy (about 200 J g^−1^) in the materials for at least 10 h at temperatures lower than the original crystallization point, unlocking opportunities for portable thermal energy storage systems.

## Abstract (web summary)

Phase-change materials offer excellent thermal storage due to their high latent heat; however, they suffer from spontaneous heat loss. Han et al., use organic photo-switching dopants to introduce an activation energy barrier which enables controllable thermal energy release and retention.

## Introduction

Phase-change materials (PCMs), such as salt hydrates^1^, metal alloys^2^, or organics^3^, store thermal energy in the form of latent heat, above their phase-transition temperature, which is released via reverse-phase transformation^4^. Long-term storage of latent heat without loss to the environment remains a challenge^5^ due to the sensitivity of phase-transition to temperature, which fundamentally prevents the deployment of thermally charged PCMs away from heat sources. One way to prevent this spontaneous heat loss is to install an energy barrier for the reverse-phase change from a high-energy phase to a low-energy phase. In some materials, intrinsic energy barriers exist, and the controlled heat release is feasible by applying external mechanical energy to overcome the barriers. For example, flexing metal clips in pockets of supersaturated salt hydrate and the application of external pressure on a ceramic material have been shown to trigger heat release from certain inorganic PCMs, but they suffer from inherent limitations. In the case of salt hydrates, the instability of the salt solution, decreasing the storage density upon cycling, and corrosiveness are the major issues that have not been resolved^6^. Ceramics, on the other hand, require a high-cost synthesis process (sintering metal oxides at *T* over 1000 °C), and possess low- energy densities (50–60 J g^−1^) compared to conventional inorganic or organic PCMs (100–600 J g^−1^)^7^. Moreover, mechanical triggering for both cases is limited by the high cost for large-scale applications.

Organic phase-change materials, such as low-cost paraffin waxes^8^, fatty acids^9,10^, polyethylene glycols^11^, and sugar alcohols^12^, generally exhibit large latent heat and solid–liquid phase transitions, covering a wide range of melting and crystallization points^13^. Since the phase changes are governed by intermolecular interactions, including van der Waals, dipolar, and hydrogen bonding, the phase- transition temperatures and thermal energy densities can be controlled by tuning these key interactions between constituents.

Organic photoswitches that undergo reversible structural changes upon light irradiation have been integrated into various materials for applications, including light-driven actuation, drug delivery, sensing, optical memory, and so on^14,15^. Among photochromic molecules, such as spiropyran^16,17^, azobenzene^18–21^, fulvalene diruthenium^22^, and dithienylethene^23,24^, azobenzene has been widely explored due to its well-known shape (molecular length of 9 Å for *trans*, 5.5 Å for *cis*; planar geometry in *trans*, the benzene ring tilted at 56° from the other ring in *cis*)^25^ and polarity changes (dipole moment of 0−1.2 D for *trans*, 3.1−4.4 D for *cis*)^26^ upon photoisomerization. The planar-to-twisted conformational change of azobenzene upon UV irradiation and the reverse isomerization triggered by visible-light illumination can be utilized to alter the physical properties of surrounding molecules through the change in intermolecular interactions.

Herein, we introduce azobenzene dopants into conventional organic PCMs as a way to change the intermolecular dynamics. These dopants, possessing activation energy barriers for switching between photoisomers, provide stability to the phase storing thermal energy and triggerabilty for energy release, thus allowing controllable, high-density energy storage in scalable organic composites. Specifically, the azobenzene dopants that change conformation upon illumination can be locked in the liquid phase of PCMs by lowering their crystallization temperature (*T*_c_), retaining the thermal energy storage at cooler temperatures.

## Results

### Thermal energy storage and release in PCM composites

We prepared a composite of tridecanoic acid, as an example of n-fatty acids with high heat of fusion (177 J g^−1^), and an azobenzene dopant that is functionalized with a tridecanoic ester group to render high miscibility with the PCM molecules. Long aliphatic compounds such as n-fatty acids or n-paraffin waxes form lamellar structures through hydrocarbon side packing (i.e., van der Waals forces), and the CH_3_ end-group interaction between the lamellae can affect the molecular arrangement as well, leading to polymorphism^27^. In the case of n-fatty acids with –COOH groups, the polar interaction and H-bonding between the acid groups can also impact the lamellar formation^28^. The azobenzene dopants possess strong *π–π* interactions^29^ among adjacent aromatic cores and van der Waals interactions between alkyl chains. Ester linkers are also expected to contribute to intermolecular interactions.

**Fig. 1:**
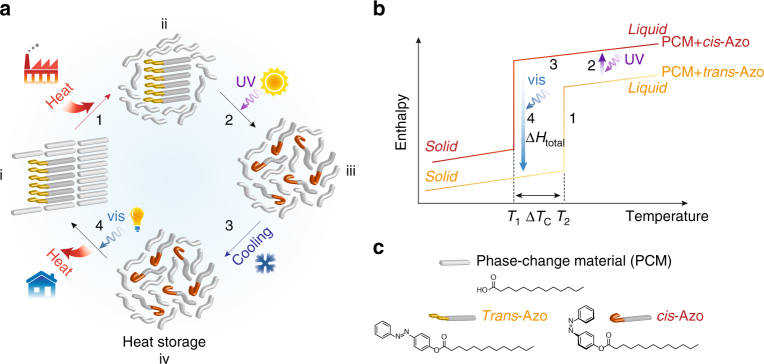
Optically controlled thermal energy storage and release cycle. **a** Schematic of (1) thermal energy absorption by phase-change materials (PCM) composite, (2) ultraviolet (UV) illumination for photoisomerization of azobenzene dopants, (3) cooling of liquefied PCM composite, and (4) visible-light (vis)-triggered reverse isomerization and heat release. The straight and curved rods exhibit different degrees of translational and rotational freedom, i.e., different phases. **b** Schematic energy diagram of PCM composites containing uncharged (*trans*-Azo) or charged (*cis*-Azo) dopants, showing enthalpy and temperature changes along (1)–(4) stepwise process for the thermal energy storage and release cycle. *T*_1_ and *T*_2_ are crystallization points of charged and uncharged composites, respectively, and Δ*T*_c_ is the difference between *T*_1_ and *T*_2_. Δ*H*_total_ represents the expected heat release from the optically discharged composite. **c** Chemical structures of PCM (tridecanoic acid) and the azobenzene dopant, functionalized with tridecanoic ester group, in each isomeric form

The first step in the thermal storage cycle is the absorption of external thermal energy by the solid composite that is crystalline as prepared (Fig. 1a, i). When heated above the melting point (*T*_m_) of the PCM (42 °C), the composite becomes a mixture of molten PCM and crystalline aggregates of the azobenzene dopant, which has a higher melting point of 73 °C (Fig. 1a, ii). Then UV illumination of the slurry switches the *trans* azobenzene dopants into *cis*, and the resulting *cis*-dopants with a twisted conformation become well dispersed in the liquid PCM (Fig. 1a, iii). We note that the temperature of the composite is maintained to be above 42 °C during the UV-charging process, which is enabled by simultaneous heating and UV absorption processes (see Methods section). This liquid composite is storing the fractional latent heat of the PCM (177 J g^−1^), that of *trans* azobenzene dopants (118 J g^−1^), as well as the fractional isomerization energy of the metastable *cis* azobenzene (116 J g^−1^). Surprisingly, the liquid state of the composite can be conserved through subsequent cooling to a temperature below the original *T*_c_ (38 °C), while the latent thermal energy stored is fully maintained (Fig. 1a, iv). This striking heat storage ability of the composite is achieved by the metastable *cis*-dopants that can disrupt the packing of PCM molecules through steric repulsion and dipolar interactions, and require triggering to overcome the activation barrier for reverse isomerization to their ground-state *trans* form. Visible-light illumination rapidly switches the dopants and allows the PCM composite to crystallize and release the stored latent heat on-demand, recovering the original state of the composite (Fig. 1a, i).

The PCM composites before and after UV/thermal charging possess different phase-transition temperatures and scales of latent heat (Fig. 1b). Enthalpy and temperature changes, during the thermal storage and release cycle, are depicted as follows: (1) rise of temperature and enthalpy during the PCM melting, (2) isothermal enthalpy increase by UV illumination, (3) temperature and enthalpy decrease during cooling to an arbitrary temperature between *T*_1_ and *T*_2_, and (4) visible-light-triggered isothermal exothermic reaction. Δ*T*_c_, a figure of merit in this system, represents the degree of phase stabilization of the charged composite (PCM + *cis*-Azo) without losing heat. Δ*H*_total_, the expected exothermic energy density from the system, should be comparable to that of the pristine PCM for high-performance thermal storage applications. Figure 1c shows the basic structures of the PCM and the azobenzene dopants, selected for this proof-of-concept study, and we also explored other dopant derivatives that are systematically functionalized on the *para*-position of the azobenzene core to alter the intermolecular interactions in the resulting composites.

### Optical control of PCM composite phase

**Fig. 2:**
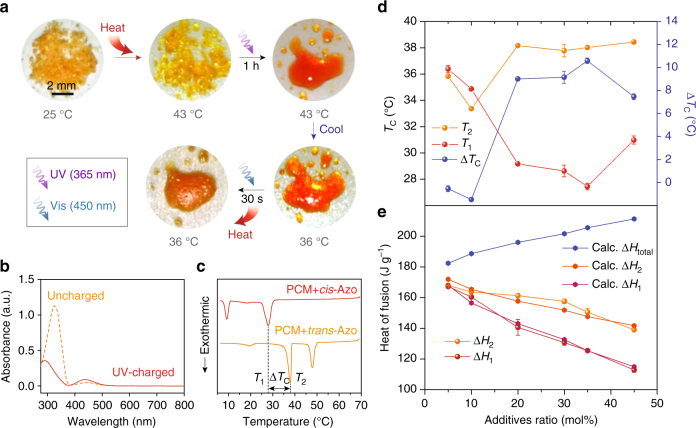
Experimental observation of phase change and heat storage in composites. **a** Photographs of composites of tridecanoic acid and 30 mol% of azobenzene dopants during solid-state heat absorption/ UV charging/cooling/visible-light-induced discharging and heat-release process. **b** UV-vis absorption spectra of the azobenzene dopant in dichloromethane solution, before and after UV illumination. The uncharged solution is saturated with *trans* isomers (dotted yellow line), showing peak absorption at 325 nm (*π* → π* transition), and the UV-charged solution saturated with *cis* isomers (solid red line) exhibits a *n* → π* transition peak at 440 nm^48,49^. **c** Differential scanning calorimetry (DSC) scans of charged and uncharged composites (35 mol% doped) obtained while cooling from 70 °C at a rate of 5 °C min^−1^, illustrating different crystallization points (*T*_1_ and *T*_2_) and the gap (Δ*T*_c_). Small peaks at 20 °C indicate the solidification of a minor polymorph^50^ in tridecanoic acid. **d** *T*_1_, *T*_2_ (left axis), and Δ*T*_c_ (right axis) measured by DSC at a rate of 5 °C min^−1^ from composites with varying additive ratios. **e** Heat of fusion (i.e., crystallization enthalpy) measured by DSC at a rate of 5 °C min^−1^ and calculated on the composites with varying additive ratios. Error bars in **d**, **e** indicate standard deviations of the data (either temperature or heat of fusion) collected at least 5 times on each type of composite

Consistent with the schematic cycle shown in Fig. 1, the crystalline composite was partially molten when heated above the *T*_m_ of the PCM, then charged by a UV (365 nm) lamp to become fully liquid, then able to be cooled below the *T*_c_ of the pure PCM (38 °C), and was finally discharged by a blue (450 nm) LED light (Fig. 2a). The azobenzene dopant exhibits typical optical absorption properties of the *trans* and *cis* isomers, before and after UV illumination in solution (Fig. 2b)^30^, and the relative ratio of each isomer in the uncharged compound (94% *trans*) and in the UV-charged compound in the photostationary state (94% *cis*) was measured by^1^H NMR (Supplementary Fig. 1a, b). The efficiency and kinetics of solid-state *trans* → *cis* conversion of the azobenzene dopant in the PCM composites was also analyzed by ^1^H NMR (Supplementary Fig. 1c) which indicates the saturation of *cis* isomers at 90% after 1 h of charging. The dispersion of *cis* azobenzene in liquid PCM facilitates the uniform charging of the composite, and the time scale estimated for the dispersion is consistent with the experimental time scale for *cis* saturation (see Supplementary Note 1). *T*_c_ of the charged composite was measured by differential scanning calorimetry (DSC), and compared to the uncharged composite (Fig. 2c). The charged composite crystallizes at 28 °C, followed by the crystallization of *cis*-Azo at 9 °C, while the uncharged counterpart exhibits the first solidification peak of *trans*-Azo at 48 °C, followed by the PCM crystallization at 38 °C. We note that the crystallization temperature of *trans*-Azo (60 °C measured as a pristine material) is significantly lowered in the PCM composites, as a result of the solvation by the liquid PCM molecules at temperatures higher than the PCM crystallization point. The melting point of *trans*-Azo (originally 73 °C) is also lowered, due to the solvation effect, and the degree of the changes in phase-transition temperatures can be variable, depending on the doping level in the composite and cooling rate.

Interestingly, *T*_1_ and *T*_2_ change significantly at varied doping levels (Fig. 2d) with the maximum ∆*T*_c_ obtained at a doping level of 35 mol%. At low-doping levels (5–10 mol%), both *T*_1_ and *T*_2_ decrease slightly from the original *T*_c_ of the PCM by a few degrees, resulting in negligible ∆*T*_c_ (Supplementary Fig. 2a). Above 20 mol% doping, however, *T*_2_ remains almost unchanged from 38 °C, and the azobenzene dopants crystallize separately at higher temperatures, as seen in Fig. 2c. ∆*T*_c_ generally increases with a higher doping level but decreases at a doping level of 45 mol%. *T*_2_ is fixed at 38 °C while *T*_1_ increases up to 31 °C, and the charged composite shows a minor exothermic peak at 36 °C which can be assigned to the solidification of the *trans*-Azo dopant (Supplementary Fig. 2b). The relative crystallization enthalpy of the charged (Δ*H*_1_) and uncharged (Δ*H*_2_) composites was obtained by integrating the crystallization peaks (Fig. 2e). Δ*H*_1_ and Δ*H*_2_ are the sum of crystallization enthalpies of the PCM (∆*H*_PCM_) and dopant (∆*H*_cis-Azo_ or ∆*H*_trans-Azo_) at relative weight fractions (χ between 0 and 1).

1 $${\rm{\Delta }}{H_1} = \chi {\rm{\Delta }}{H_{{\rm{PCM}}}}{\rm{ + }}\left( {{\rm{1}} - \chi } \right){\rm{\Delta }}{H_{{\rm{cis}} - {\rm{Azo}}}}$$

2 $$\Delta {H_2} = \chi {\rm{\Delta }}{H_{{\rm{PCM}}}} + \left( {1 - \chi } \right){\rm{\Delta }}{H_{{\rm{trans}} - {\rm{Azo}}}}$$

The measured Δ*H*_1_ and Δ*H*_2_ are well aligned with the calculated values, indicating that the dopants are mobile in the PCM medium and prone to aggregation, regardless of the isomeric state due to the strong dopant–dopant interactions. ∆*H*_total_ is the heat released from liquid (*cis*) → solid (*trans*) transition of the composite, so it is described as

3 $$\Delta {H_{{\rm{total}}}} = \chi {\rm{\Delta }}{H_{{\rm{PCM}}}} + \left( {1 - \chi } \right){\rm{\Delta }}{H_{{\rm{trans}} - {\rm{Azo}}}} + \left( {1 - \chi } \right){\rm{\Delta }}{H_{{\rm{iso}}}}$$

∆*H*_iso_ is the isomerization enthalpy of the azobenzene dopant, measured by DSC during thermal reverse isomerization (*cis* → *trans*) occurring in the liquid state (Supplementary Fig. 3a), and ∆*H*_iso_ of 116 J g^−1^ (46 kJ mol^−1^) is comparable and slightly improved from that of pristine azobenzene (41.4 kJ mol^−1^)^31^. A quantitative measurement of Δ*H*_total_ was challenging due to the difficulty in decoupling the heat release from the composite and the heat absorption from the light source for optical triggering during the DSC measurement. However, assuming the complete *cis* → *trans* conversion, the expected heat release is considerable and increases with higher doping levels. When highly doped, ∆*H*_PCM_ decreases, while the contributions of ∆*H*_trans-Azo_ and ∆*H*_iso_ increase. If it is 100% doped, the pristine azobenzene dopant has a potential to release 234 J g^−1^ of heat, but the requirement of heating it above its high *T*_m_ (73 °C) for the solid-state charging induces the thermal reverse isomerization of the *cis* isomer, preventing the control over thermal storage. Also, UV illumination of azobenzene in the solid state without heating is ineffective as a result of steric confinement of aromatic groups in the crystalline lattice^32^. Thus, the design of PCM and the azobenzene composite has a unique advantage to realize thermal storage at temperatures above the relatively low *T*_m_ of the PCM.

**Fig. 3:**
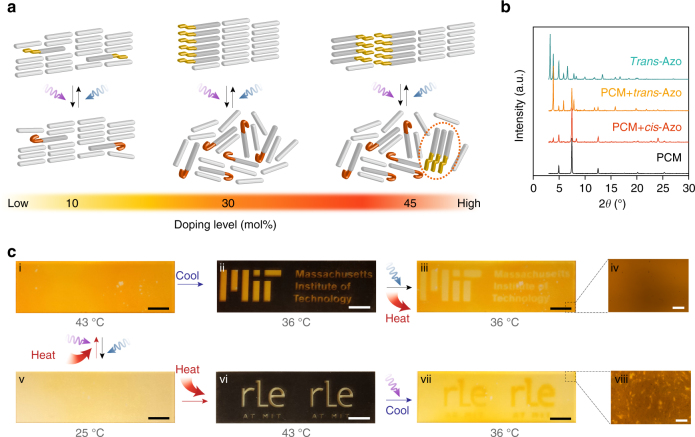
Proposed mechanism and demonstration of the optical manipulation of composites. **a** Expected distribution and arrangement of azobenzene and phase-change materials (PCM) molecules at room temperature when dopants are uncharged and charged by ultraviolet (UV) illumination, at varied doping levels. **b** X-ray diffraction patterns of *trans* azobenzene dopant (*trans*-Azo), PCM, and the composites with 30 mol% charged (PCM + *cis*-Azo) and uncharged dopants (PCM + *trans*-Azo), obtained at room temperature. **c** Demonstration of reversible charging and discharging of PCM composite films (30 mol% doped), sandwiched between two glass slides. (i–iii) UV/thermally charged composite was cooled at 36 °C and illuminated with visible light selectively on the uncovered area (orange letters), while the rest of the film was covered by a black mask under the isothermal condition. Scale bar is 10 mm. (v–vii) Uncharged composite was heated, and only the uncovered area (yellow letters) was irradiated with UV for 1 h. Then, the sample was cooled at 36 °C. Scale bar is 10 mm. Optical microscopic images of a charged part (iv, liquid) and discharged part (viii, crystalline solid) of composite films. Scale bar is 100 µm

The dependence of Δ*T*_c_ and Δ*H* on the doping level can be interpreted as a result of different degrees of nucleator formation and supercooling (Fig. 3a). At the low-doping level (5–10 mol%), both *trans* and *cis* dopants are well dispersed in the PCM and lower the *T*_c_ of the composite by similar amounts, thus making Δ*T*_c_ negligible (Fig. 3a, left). The low-doped composites are eutectic, showing no phase separation of PCM and dopants during solidification. However, with increased doping (20–35 mol%), the photo-switching action can influence Δ*T*_c_ considerably due to the drastic differences in the aggregation dynamics of charged and uncharged dopants (Fig. 3a, center). The *trans* dopants first aggregate while the PCM composite is cooled and play a role as nucleating agents that facilitate the crystallization of PCM molecules on their surface. The addition of nucleators in the conventional PCMs for suppression of supercooling is well known, and the requirements for effective nucleators (high *T*_m_, an isomorphous structure to the PCM)^33,34^ are fulfilled by the properties of the *trans* azobenzene dopant aggregates. In contrast, *cis* dopants have strong interactions with the polar PCM molecules as well, leading to significant supercooling in the absence of nucleators, and the values of Δ*T*_c_ are as high as 10 °C. If more dopants (45 mol%) are present, however, the solid-state UV charging of the composite is incomplete due to the relatively low fraction of the available molten PCM for solvating the *trans*-Azo dopants by overcoming the strong dopant–dopant interactions. (Supplementary Fig. 4) Therefore, the unconverted *trans*-Azo aggregates in the charged composites, although a minority of the total concentration, can act as nucleators and reduce the degree of supercooling and Δ*T*_c_ (Fig. 3a, right).

X-ray diffraction patterns (Fig. 3b) show the prominent aggregation of *trans* dopants in the composites. The pattern of the uncharged composite shows both a distinct PCM and *trans*-Azo signatures, while that of the charged composite exhibits the major peaks of PCM and almost negligible peaks of *trans*-Azo that remains in the composite after solid-state charging (Supplementary Fig. 1c). Selective optical charging and discharging was conducted on composite films (Fig. 3c). The demonstration of local crystallization and liquefaction of the composite confirms that optical manipulation of the azobenzene conformation is indeed the key to phase transformation and thermal energy storage in the composites. The patterns can be easily removed by either UV/thermal charging for 1 h or by visible-light discharging for 30 s, and can be recycled repeatedly.

### Intermolecular interactions controlling the phase change

**Fig. 4:**
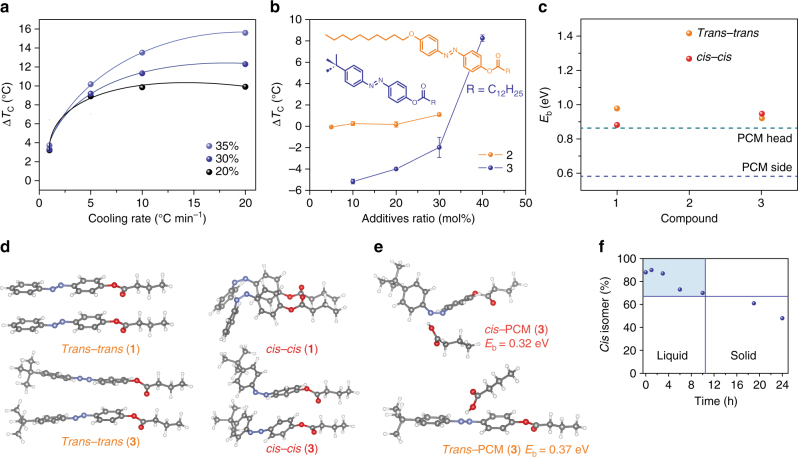
Influence of measurement conditions and dopant structures on thermal storage properties. **a** Impact of cooling rate on ∆*T*_c_ at varied doping levels. ∆*T*_c_ generally increases with higher cooling rates, and was measured by DSC through heating and cooling between 10 °C and 70 °C. **b** Impact of functionalization of azobenzene dopants on ∆*T*_c_. Compound **2** is functionalized with a long alkyl chain (decyloxy group), and compound **3** is decorated with a bulky substitution (*tert*-butyl group). Error bars indicate standard deviations of temperature measured at least 5 times on each type of composite. **c** DFT-calculated binding energy between two neighboring molecules (either as *trans*/*cis* isomer or PCM) varying with the functional group on the *para*-position of the azobenzene core. Dotted lines represent the head-to-head interaction between acid groups of the PCM (green) or the side-by-side interaction between alkyl chains of the PCM (blue). **d** DFT-simulated configurations of two *π*–*π* stacking dopants (compound **1** and **3**) as *trans* or *cis* isomer. **e** DFT-simulated configurations of a dopant (compound **3**, *cis* or *trans*) and a neighboring PCM molecule interacting around N=N group of azobenzene and –COOH group of PCM. **f** Stability of heat storage in a UV-charged liquid composite (30 mol% of compound **1**) in the dark at 36 °C. The thermal reverse conversion of azobenzene dopants (*cis* → *trans*) was monitored by ^1^H NMR (half-life of 24 h). For 10 h (with more than 65% *cis* fraction), the composite remains a liquid, conserving heat (shaded area). After 10 h, PCM molecules gradually solidify, and the composite loses the stored heat over time under isothermal conditions

In addition to dopant concentration, the cooling rate of UV/thermally charged composites also influences Δ*T*_c_, impacting *T*_1_ more than *T*_2_. *T*_2_ is fixed around 38 °C, due to the facile formation of *trans*-Azo nucleators, while *T*_1_ decreases significantly at fast cooling rates (Fig. 4a). When slowly cooled, the diffusion of *cis* dopants and the *cis* → *trans* conversion can reduce the impact of the *cis* dopants on disrupting the PCM arrangements, resulting in a higher *T*_1_. In particular, the heating and cooling between 10 and 70 °C at the slowest rate of 1 °C min^−1^ exposes the metastable *cis* isomers to high-enough temperatures to enable thermal reverse isomerization over a considerable period of time, which was confirmed by the observation of *trans*-Azo aggregates through DSC measurements, leading to underestimated ∆*T*_c_ values (3–4 °C).

Beyond concentration and the cooling rate, the chemical structures of photo-switching dopants and the relative strength of intermolecular interactions between dopants and that with PCM molecules can vary Δ*T*_c_ in remarkably different ways (Fig. 4b). Compound **2** is a derivative of compound **1** with a decyloxy group substituted on azobenzene at the *para*-position, designed to increase the van der Waals interactions between alkyl chains present in both the PCM and dopants. Compound **2** exhibits the highest *T*_c_ of 68 °C, indicating strong binding forces between the dopants through π–π stacking of aromatic cores and side packing between alkyl chains. Yet, compound **2** was observed to have a negligible impact on Δ*T*_c_ compared to compound **1**, because of the inefficient solid-state charging of *trans* aggregates in the PCM composites due to their strong packing (Supplementary Fig. 4). The charged composites show both *trans* and *cis* dopant crystallization peaks (around 60 and 10 °C, respectively), and the ratio of the *trans/cis* peak intensity increases with higher doping levels, confirming the difficulty in solid-state charging of strong aggregates.

Compound **3**, on the other hand, possesses a bulky *tert*-butyl substitutent that significantly diminishes the *π–π* interactions between aromatic groups, helping to prevent the formation of nucleating agents in the composites. Interestingly, due to the sterically hindering nature of *tert*-butyl groups, the *trans* dopant can effectively disrupt the PCM alignment, even better than the *cis* isomer, as the negative value obtained for Δ*T*_c_ implies. The *cis* dopants exert a similar degree of supercooling (by 5–6 °C) as the *cis* form of compound **1** at a doping level of 10 mol%, but the *trans* dopants induce a much larger supercooling (by 8–13 °C, over varied cooling rates). When the low numbers of dopants are dispersed in the PCM, the interactions between a dopant and the surrounding PCM molecules become the predominant factor that predicts the crystallization dynamics. However, at higher doping levels, dopant–dopant interactions and aggregations become increasingly important, as the formation of nucleators can greatly reduce the supercooling. Despite the repulsion between *tert*-butyl groups, the *trans* isomers crystallize at 44 °C (Supplementary Fig. 3b), whereas the *cis* isomers remain in the liquid state upon cooling down to −20 °C (Supplementary Figs. 3c, 5 and 6; Supplementary Movies 1 and 2)^35^, implying very little chance of forming any nucleator in the cooled composites. Indeed, the UV-charged composites are increasingly supercooled with more dopants, and the uncharged composites show a more slowly increasing degree of supercooling, competing with the nucleation process. At a doping level of 30 mol%, *trans* and *cis* isomers induce similar levels of supercooling, resulting in Δ*T*_c_ closer to zero and similar Δ*H*_1_ and Δ*H*_2_ values (Supplementary Fig. 7). At 40 mol% doping, Δ*T*_c_ is found to be positive, and Δ*H*_2_ exceeds Δ*H*_1_, as was the case for compound **1**. The X-ray diffraction patterns (Supplementary Fig. 8) of low-doped (10 mol%) and high-doped (40 mol%) composites also corroborate these findings as follows: (1) higher impact of *trans* dopants on the PCM packing than that of *cis* isomers at a low level of doping and (2) larger disruption of PCM alignment by *cis* dopants at a high doping level.

We explored the binding energy (*E*_b_) between two molecules (either dopant or PCM) via ab initio calculations, and found that the dopant–dopant interaction is generally stronger than the PCM–PCM interaction, which explains the facile solidification of dopants in composites during the cooling process (Fig. 4c). We note that the binding energies of the *cis* dopants are generally overestimated due to the neglect of finite-temperature effects and ensemble environment. *E*_b_ of compound **2** is much larger than that of other dopants, justifying its stronger tendency to aggregate and the negligible Δ*T*_c_ of the composites. Consistent with the greater probability to form *trans* rather than *cis* aggregates, the binding between *trans* isomers is stronger than that between *cis* isomers for compound **1** and **2**, even though the dynamic effects that may further reduce the stability of the *cis* crystal were not accounted for in the simulations. Upon functionalization with the *tert*-butyl group (compound **3**), *E*_b_ decreases in the *trans* while it increases in the *cis* dimer (configurations shown in Fig. 4d, compared to compound **1**), which is in agreement with the higher concentration required for *trans* dopant aggregation observed in the experiment. In the low-doping region, both *trans* and *cis* dopants are dispersed, and thus, the interaction between an isolated dopant and the surrounding PCM molecules becomes the dominant factor that governs the crystallization dynamics. This situation was analyzed by considering *E*_b_ between a *trans* isomer and a truncated PCM molecule in various energetically favorable configurations (Fig. 4e). In addition to the strongest binding between –COOH and ester groups, the results suggest that bound states are also feasible via the formation of hydrogen bonds with N atoms or van der Waals interactions between the –COOH group and aromatic rings (Fig. 4e). Compared to *cis*, an isolated *trans* may have a higher impact on disrupting PCM arrangement due to the planar structure that provides a larger space for binding on many different sites. The *cis* isomer, with such a bulky substituent, limits the interaction with another PCM molecule, for example, on the back side of the N = N group (the opposite configuration to Fig. 4e, top) because of the significant steric repulsion.

Finally, the stability of thermal energy storage in a UV/thermally charged composite (30 mol% of compound **1**) was measured by ^1^H NMR analysis of the *cis* fraction and by monitoring solidification of the composite over a period of 24 h in the dark at the cooled temperature (36 °C, 6 °C below the *T*_m_) (Fig. 4f). The half-life of the *cis* dopant in such conditions was about 24 h, and the liquid phase was conserved for at least 10 h. When the *cis* ratio drops from 70% (10 h) to 65% (19 h), the solidification of the PCM was observed, which was also consistent with optical triggering experiments (Supplementary Fig. 9).

### Energy efficiency analysis

The concept of this study is fundamentally different from that of conventional solar thermal fuels (STFs)^36–39^, or molecular solar thermal (MOST) systems^22,40^, which convert photon energy into stored thermal energy. Instead, here, we demonstrate a unique approach that uses the photon energy as a way to control the phase-transition properties of traditional thermal storage materials such as PCMs. The energy efficiency of this type of energy-storage system will depend on the thermal energy input from a high-temperature heat source (Δ*H*_2_) and the released thermal energy at a lower temperature upon optical triggering (Δ*H*_total_). As described in equation (3), Δ*H*_total_ is the sum of Δ*H*_2_ and the reverse isomerization energy of azobenzene dopants (*cis* → *trans*), making it a greater value than the thermal storage density of each component (i.e., PCM molecule or the azobenzene dopant).

We note that the photon energy required for operating an azobenzene switch is considerable, given the low quantum yield (ca. 10%, different depending on the measurement conditions^41,42^) of its photoisomerization (*trans* → *cis*). For example, the PCM composite containing 30 mol% of azobenzene dopants requires UV (365 nm) photon energy of ca. 3.6 kJ per 1 gram of the composite for the photoinduced phase fixation, while the total heat storage in 1 gram of the composite is ca. 0.2 kJ, or about 5–6% of the input photon energy. This may seem to be inefficient, but the photon energy consumption for this system is significantly lower than that for conventional STFs. The energy-storage capacity of pristine azobenzene as an STF is 0.23 kJ g^−1^ ^31^, while the input UV (365 nm) photon energy density is 18.0 kJ g^−1^ (1.3% efficiency). Other azobenzene derivatives that exhibit higher energy storage as a result of structural designs provide slightly improved efficiencies such as 2.7%^32^ and 3.0%^43^, assuming approximately 10% quantum yield. Utilizing azobenzene photoswitches as a minor component in the PCM composite gives rise to the relatively low demand for photon energy input, compared to that for STFs that consist of 100% photoswitches. Both STFs and photoswitchable PCMs would benefit from further development of high-quantum-yield photo-switching molecules to increase the energy efficiency. However, we note that abundant solar energy and radiation are expected to replace the current UV lamps, used for the experiments at this stage, as demonstrated on STFs by Saydjari and coworkers^44^.

We also note that this approach is fundamentally distinguished from the use of thermal insulation that is currently used for decreasing the cooling rate of thermally activated materials. While thermal insulation can reduce the loss of sensible heat, it cannot stop the spontaneous heat transfer between the thermally charged PCMs and the cooler surroundings (see Supplementary Fig. 10 for schematic energy diagrams comparing the two concepts) or the liquid-to-solid phase transition which leads to the loss of latent heat. In contrast, the photoswitchable PCM systems, which are able to still provide thermal storage even in the absence of thermal insulation, effectively preserve latent heat by changing the intrinsic properties (i.e., crystallization temperature) of the PCMs. As with traditional PCMs, thermal insulation can also be used with the photoswitchable PCMs to slow down the loss of sensible heat, but the advantage is the ability to hold on to the latent heat until it is released. Another difference between the two approaches is the triggerability of heat discharge. Optical triggering of the latent heat release from the photoswitchable PCMs is a newly introduced property to pristine PCMs with or without thermal insulation.

Based on these differences, we can compare the energy efficiency of azobenzene-doped PCMs and that of conventional PCMs with thermal insulation (Supplementary Note 2). As a result of gradual sensible heat loss through an insulation layer, conventional PCMs need external thermal energy input to maintain the liquid state at a high temperature, which leads to the efficiency drop over time (Supplementary Fig. 11). On the other hand, the efficiency of azobenzene-doped PCMs is determined by the UV photon energy input during the initial charging process that fixes the liquid state in the dark until a critical percentage of *cis* azobenzene undergoes reverse isomerization. As aforementioned, the use of photoswitches with higher quantum yields will significantly increase the energy efficiency that is constant over time in this system.

## Discussion

The goal of our work is to present a unique method to modify the thermodynamic properties of conventional PCMs and to explore the design criteria of the photo-switching dopants. For the potential applications and practical devices, multiple aspects of the system need to be optimized, and the potential challenges should be addressed. First, we envision a device structure that involves a container with windows and covers needed for the controlled light absorption and dark storage. Thermal energy from various waste heat sources, including solar heat, is indirectly transferred to the PCM composite using a heat exchanger, and a separate UV source (an LED or a gas-discharge lamp) will be used for the simultaneous azobenzene-charging process. The thermally activated PCM will be simply carried to a heat outlet at room temperature with the covers closed to enable dark storage. To trigger heat release, the covers will be opened to expose the liquid composite to ambient light or blue LED light for a faster release.

The PCM composite is designed to keep the stored latent heat even when it is cooled down to room temperature. Therefore, the facile heat loss through the glass windows or through the container without thermal insulation is considered as a part of the heat-transfer process to reach equilibrium with the cooler surroundings, while it would be a challenge in the pristine PCM system that solidifies at the original crystallization point. The common borosilicate glass that allows over 90% transmission of 365-nm UV and visible light will be suitable as the window material.

The implementation of a stirring or flowing system in the container can solve multiple potential challenges associated with the UV charging and visible-light discharging processes, including the fixed penetration depths of light, the initial phase separation of PCM and *trans*-Azo (during UV charging), and the scattering of visible light by the solidified portion of the PCM (during discharging).

In our system, the azobenzene dopants are suspended in the viscous liquid PCM when they are irradiated with UV, and solvated by the PCM as *trans*-to-*cis* conversion occurs. The dopants can diffuse in the viscous liquid phase of the PCM, which enables the facile charging of thick samples, similar to the complete charging of azobenzene solutions (in dichloromethane) in 20-mL vials that are 23-mm thick. The powder samples as shown in Fig. 2a are 100–200-μm thick, and the azobenzene dopants are successfully charged by UV irradiation (ca. 90%, Supplementary Fig. 1c). This indicates that the dynamics in the composite play an important role in increasing the actual penetration depth, despite the smaller calculated static penetration depth (23 µm, see Supplementary Note 3, Supplementary Fig. 12, and Supplementary Movie 3 for a detailed analysis). Therefore, we envision that the liquid PCM composite, once equipped with a stirrer or a flow system, will not be limited by the static light penetration depth or the initial phase separation, due to the uniform exposure to UV and the facile dissolution of *trans*-Azo aggregates. The stirring or flowing system will also effectively remove the scattering layer and disperse the nucleating sites in the bulk PCM during the discharging step, which allows for facile crystallization and propagation in a large-scale composite.

**Fig. 5:**
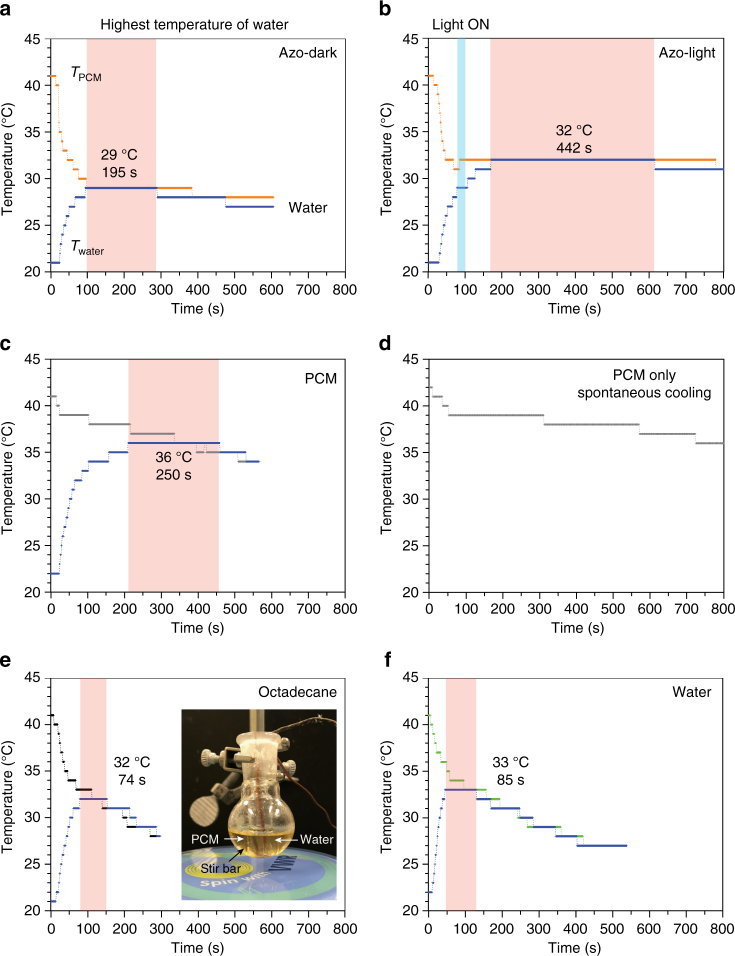
Water-heating experiments. Showing the temperature changes of 1 g of water (blue curve) in contact with 3 g of **a**, ultraviolet-charged phase-change materials (PCM) composite (35 mol% of *cis* azobenzene dopant (compound **1**)) in the dark. **b** Optically triggered composite by the exposure to a blue light-emitting diode lamp at 31 °C for 500 s to maximize the discharging of *cis* azobenzene, (**c**) pristine PCM (i.e., tridecanoic acid), (**e**) octadecane, and (**f**) water bath. The inset of (**e**) shows the setup where each thermocouple is submerged in a heating medium and in a vial containing water. The temperature of water in the vial is at 21–22 °C under ambient condition and quickly increases as the vial is immersed in the heating medium at 40 °C. The temperatures of the heating medium (*T*_PCM_) and water (*T*_water_) are recorded simultaneously after *T*_PCM_ reaches 41–42 °C while the heating fluid is stirred. **d** Temperature change of pristine PCM under ambient condition without the process of dipping a water container into the PCM heating bath

In order to demonstrate the concept at a larger scale, we devised an experiment where 3 g of a UV-charged PCM composite is optically triggered to release heat which is transferred to 1 g of water and raise its temperature effectively (Fig. 5). The lowering of *T*_c_ of PCM from 38 to 29 °C by the addition of the azobenzene dopant and UV activation is clearly observed (Fig. 5a), and the triggering of crystallization above 29 °C by optical irradiation is demonstrated (Fig. 5b), in accordance with the milligram-scale DSC measurements and thin-film-patterning experiments. The heat release from the optically triggered composite is larger than that from the UV-charged composite in the dark, due to the additional contribution of dopant crystallization and azobenzene isomerization energy, consistent with our DSC measurements shown in Fig. 2. The longer crystallization process and the longer heating of water, despite the continuous and spontaneous heat loss to the cooler surroundings, indicate significant heat release from the composite and transfer to water. The impact of the blue light irradiation on the temperature of bulk materials, either PCM or water, was confirmed to be negligible, through a control experiment with pristine PCM and water. The LED lamp was placed 20 cm away from the PCM flask.

As shown in Fig. 5c, pristine PCM can heat water to a higher temperature for a shorter period, as a result of its higher *T*_c_ (38–39 °C) and the faster heat loss to the surroundings at high temperatures. The crystallization process of pristine PCM is slower without contacting to a water container (Fig. 5d). In order to emphasize the impact of phase change and latent heat on water- heating dynamics, we conducted control experiments replacing the PCM by other substances which only transfer sensible heat to water within the temperature range of study. Octadecane was chosen as a type of paraffin which has a similar thermal conductivity and heat capacity to tridecanoic acid but presents a low *T*_c_ of 28 °C (Fig. 5e), and water as a common heating medium with a high heat capacity (Fig. 5f). In both cases, the heated water cools down rapidly, in contrast to water in a solidifying PCM composite bath, demonstrating the significant advantage of PCMs over heated liquid as a heating medium. Over 5 times longer heating of water enabled by the light-triggered phase change than by the sensible heat transfer from other heated fluids shows the significance and practicality of latent heat storage and release.

Finally, the long-term stability and reversibility of the photo-switching composites needs to be addressed. High thermophysical stability of the azobenzene dopant (compound **1**) at temperatures up to 200 °C and the optical cycling stability over 100 cycles of charging and discharging for over 50 h were probed (Supplementary Fig. 13), and the invariable morphology of the composite after optical discharging was analyzed by the Fourier transforms of optical microscope images (Supplementary Fig. 14). Further X-ray diffraction studies, however, indicate that optically induced fast crystallization can lead to less aggregation and incomplete *cis*-to-*trans* conversion, consistent with the molar fraction analysis of the *cis* isomer during optical discharging (Supplementary Fig. 9). Although crystallinity of the PCM composite is slightly changed from the starting material after the initial optical discharging, the morphology and crystallinity of the composite in the subsequent cycles of UV charging and visible-light discharging will be fully reversible.

In summary, a unique method is demonstrated to control the intermolecular interactions between phase-change materials and photochromic dopants for thermal energy storage and release in the composites by optical switching. This simple approach provides the foundation to add a functionality to conventional heat-storage materials and potentially to various other types of PCMs including inorganic materials by employing suitable photoswitches that can interact with relative PCMs effectively. This proof of concept may provide insights into thermal energy management for a wide range of potential applications such as waste heat recycling, solar thermal collection, and smart temperature systems for buildings.

## Methods

### Materials

Tridecanoic acid, oxalyl chloride, and the azobenzene starting materials were purchased from Sigma-Aldrich and were used without further purification. Dichloromethane, tetrahydrofuran, dimethylforamide, and methanol were purchased from VWR and were used as received.

### Synthesis of azobenzene dopants

To a solution of tridecanoic acid (385 mg, 1.8 mmol) in dichloromethane (5 mL), oxalyl chloride (231 µL, 2.7 mmol) was added dropwise at room temperature and stirred for 10 min. A catalytic amount (a drop) of dimethylformamide was added to the mixture, and the solution was stirred for 3 h generating CO_2_ (g), CO (g), and HCl (g) through a bubbler. The reaction mixture was dried under reduced pressure (50 mTorr) for 2 h to obtain tridecanoic acid chloride (yellow oil) which was used for the next step without further purification. The acid chloride (417 mg, 1.8 mmol) was dissolved in dry THF (5 mL) which was added dropwise to the mixture of 4-phenylazophenol (534 mg, 2.7 mmol) and triethylamine (1.1 mL, 8.1 mmol) in dichloromethane (50 mL). After the gas evolution stopped, the mixture was stirred overnight, and the solvent was evaporated to a reduced volume (5 mL). Methanol (10 mL) was added to the reaction mixture to precipitate a yellow powder that was filtered, rinsed with methanol, and dried under reduced pressure. The clean product, compound **1** (567.4 mg), was obtained with 80.1% yield.

Compounds **2** and **3** were synthesized with different phenylazophenol precursors, 4-(4-decyloxyphenylazo)phenol and 4-(4-tert-butylphenylazo)phenol, and were obtained at 89.1% and 62.5% yields, respectively. The ^1^H and ^13^C NMR spectra and the structural information including HRMS of the compounds can be found in Supplementary Figs. 15–20 and Supplementary Methods.

### Sample preparation and charging/discharging procedures

The composites were prepared by dissolving the relative fractions of the PCM and azobenzene dopants mixed in dichloromethane which was evaporated at 40 °C under nitrogen flow. The solid samples were then transferred to glass substrates and were heated at 43 °C while being irradiated by a UV lamp (365 nm, 100 W) that was placed 25 cm above the samples. The UV-charging station was covered by a container and aluminum foil to block ambient light exposure. After 1 h of charging, the samples were transferred to DSC pans in the dark for measurements. For discharging, the samples were illuminated by a blue LED lamp (450–460 nm, 15 LED chips, 12 W) placed 10 cm above the samples at 36 °C for 30 s. For the patterning experiments (Fig. 3c), the composites were UV/thermally charged on glass substrates, and the liquefied samples were pressed under another glass slide to make films. For solution-state charging, the azobenzene derivatives were dissolved in dichloromethane and illuminated by the UV lamp while being stirred at room temperature. The charged solutions were then dried under reduced pressure in the dark to prepare DSC samples.

### Other measurements

^1^H and ^13^C NMR spectra were taken on Varian Inova-500 spectrometers. Chemical shifts were reported in ppm and referenced to residual solvent peaks (CD_2_Cl_2_: 5.33 ppm for ^1^H, 53.84 ppm for ^13^C, CDCl_3_: 7.26 ppm for ^1^H, and 77.16 ppm for ^13^C). Bruker Daltonics APEXIV 4.7 Tesla Fourier transform ion cyclotron resonance mass spectrometer was used for high-resolution mass determination with an electrospray ionization (ESI) source. UV–Vis absorption spectra were obtained using a Cary 60 UV–Vis spectrophotometer (Agilent Technologies) in a 10-mm pathlength quartz cuvette. DSC analysis was conducted on a Q series DSC Q20 (TA Instruments) with the RCS40 component. Powder X-ray diffraction (PXRD) patterns were recorded on Bruker D8 Discover diffractometer using nickel-filtered Cu-Kα radiation (*λ* = 1.5418 Å) with an accelerating voltage and current of 40 kV and 40 mA, respectively. Samples for PXRD were prepared by placing a thin layer of the appropriate material on a zero-background silicon crystal plate.

### Computational methods

Standard ab initio calculations within the framework of density-functional theory (DFT) were performed to optimize the geometry and calculate the binding energies of molecular dimers with various configurations, using the Vienna Ab Initio Simulation Package (VASP v5.4)^45^. Plane-wave and projector-augmented-wave (PAW)-type pseudopotentials^46^ were employed with a 400 eV kinetic-energy cutoff and the GGA-PBE exchange-correlation functional^47^. Van der Waals interactions were included by the DFT-D2 method of Grimme. A 20 Å vacuum along the direction involving π–π stacking and a 15 Å vacuum for all other directions were constructed to avoid artificial interactions between periodic images. The structures were relaxed until all forces were smaller than 0.02 eV Å^−1^.

In order to overcome the challenge of computing long molecules with a large vacuum, the truncated molecules with short tails containing only 4 C atoms were used as representatives in all simulations. This approximation is justified by considering the PCM dimers with various alkyl chains, wherein the binding energy increases linearly with increasing tail length at the rate of 0.055 eV per CH_2_ unit. Then, the final binding energies between stacking dimers as shown in Fig. 4d were obtained by the summation over the binding energy of truncated dimers and the estimated contributions from alkyl chains based on the above relation. For the exploration of the possibility to bind an individual dopant with multiple PCMs at a low-doping level (Fig. 4e), the van der Waals interactions from long tails have been excluded because most of the surrounding PCMs will not be able to ideally stack with the dopant.

### Data availability

The data that support the findings of this study are available from the corresponding author upon reasonable request.

## Electronic supplementary material

Supplementary Information

Description of Additional Supplementary Files

Supplementary Movie 1

Supplementary Movie 2

Supplementary Movie 3
