# Supplementary material for: Optically-controlled long-term storage and release of thermal energy in phase-change materials
Source: Nat Commun. 2017 Nov 13;8:1446. doi: 10.1038/s41467-017-01608-y (PMC5684416; doi:10.1038/s41467-017-01608-y)
Supplement: Supplementary file 1 — Supplementary Information [file 41467_2017_1608_MOESM1_ESM.pdf]

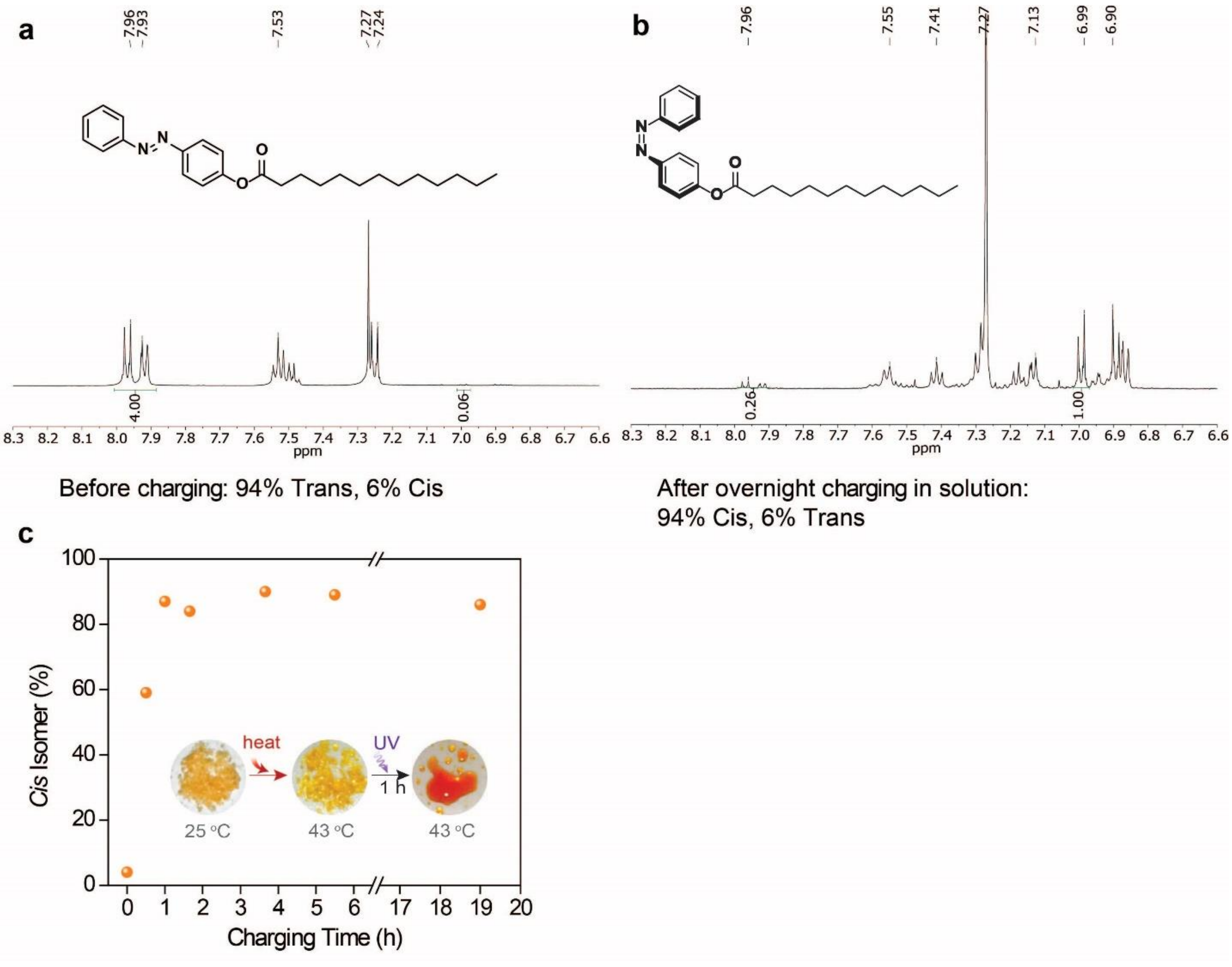

Supplementary Figure 1. ^{1}H NMR analysis of uncharged (a) and charged (b) azobenzene dopant (compound **1**) in solution via integration of peaks representing *trans* and *cis* isomers. (c) Charging of composites containing tridecanoic acid and compound **1** as dopant (30 mol%) in solid state with a UV lamp (365 nm). After UV charging, the solid composites were dissolved in solutions in dark for NMR analysis. The relative contents of *cis* and *trans* isomers were measured by the integration of NMR peaks as shown in (a) and (b). Molar fraction of *cis* isomers saturates at ca. 90% after charging time of 1 h. Inset shows the optical images of the composites during the process of solid-state charging.

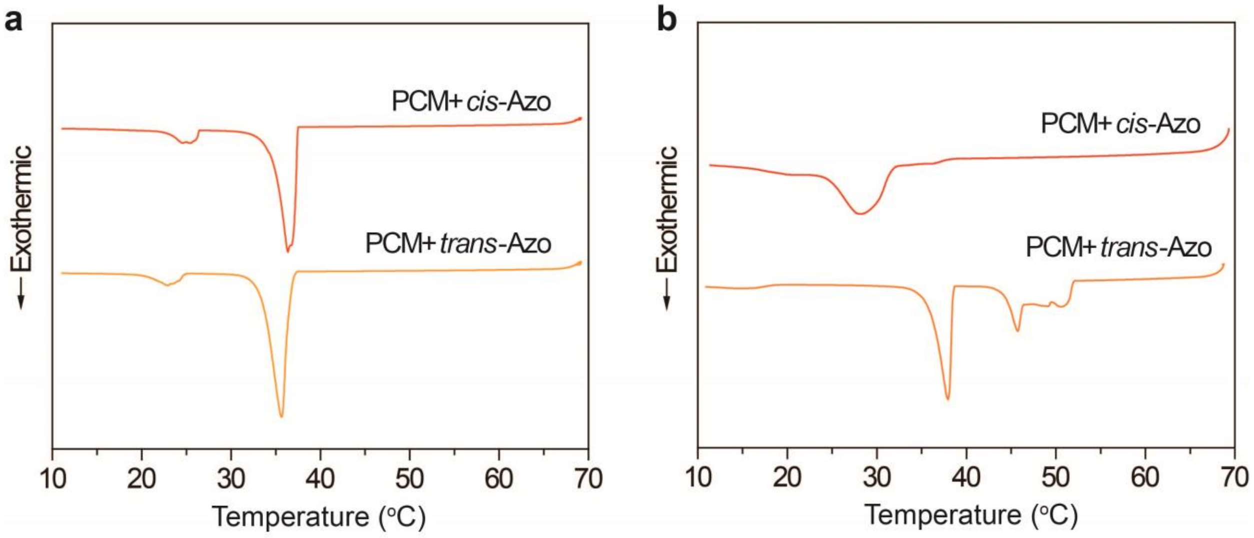

Supplementary Figure 2. DSC traces of charged and uncharged composite containing (a) 5 mol% and (b) 45 mol% compound **1** measured while cooling from 70 ^{o}C to 10 ^{o}C at a rate of 10 ^{o}C min^{-1}. (a) The uncharged composite shows congruent crystallization without separate azobenzene aggregations. The charged composite shows similar features, and the *cis*-Azo dopants crystallize at lower temperature than 10 ^{o}C (not shown). (b) There are two separate crystallization peaks of *trans*-Azo dopant and PCM, indicating the formation of nucleators by facile *trans*-Azo aggregations. For the charged composite, the minor exothermic peak (36 ^{o}C) found prior to main PCM crystallization peak (30 ^{o}C) during the cooling process is assigned to uncharged dopant aggregations (*trans*-Azo), and the *cis*-Azo dopants crystallize at lower temperature than 10 ^{o}C (not shown).

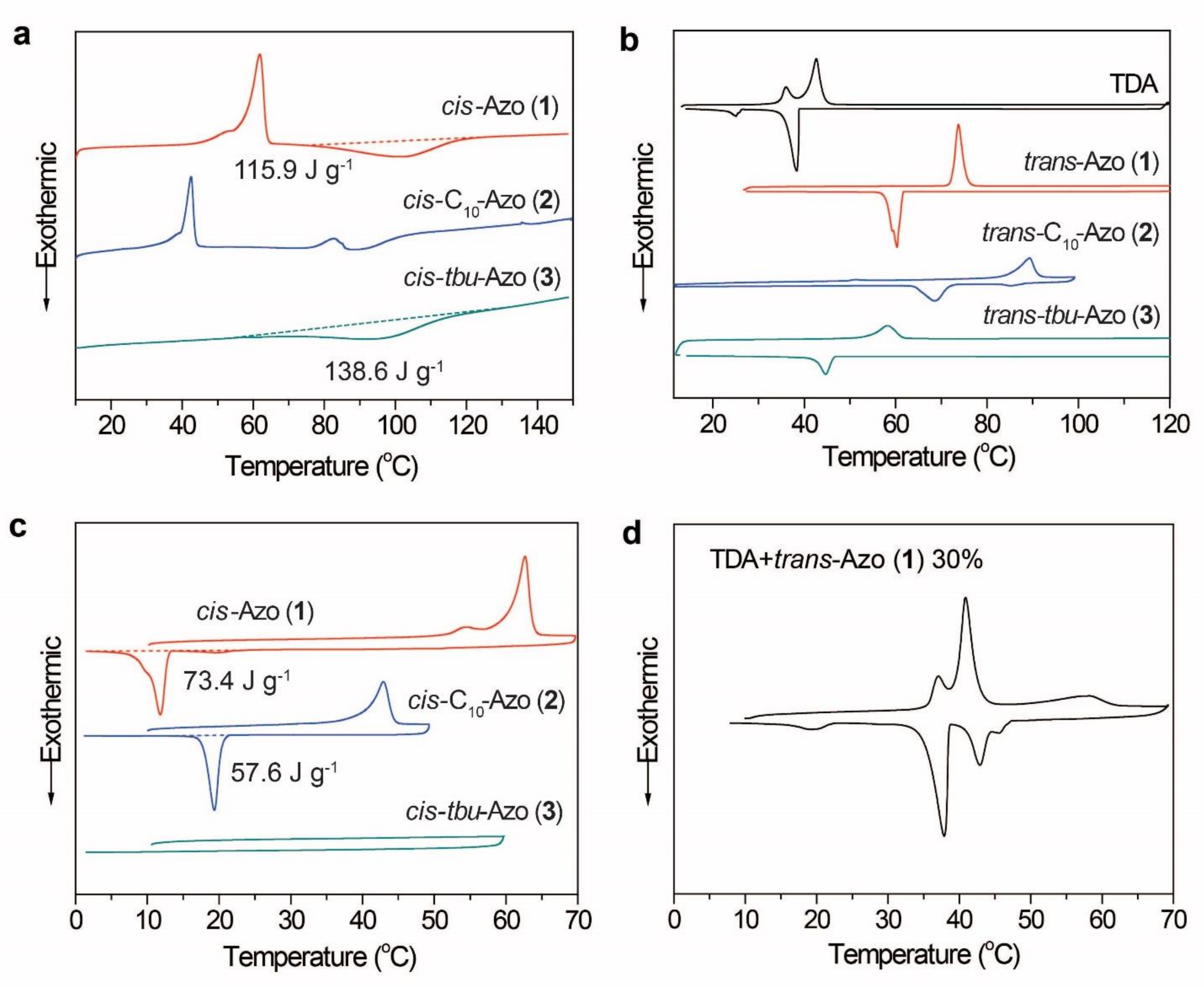

Supplementary Figure 3. DSC traces of (a) thermal discharging of charged dopants, and those of (b) uncharged and (c) charged dopants; melting and crystallization peaks are seen with charged compound **1** and **2**, but unseen with **3**. (a) Even after 24 h charging in solution, compound **2** contained minor *trans* isomers. As a result of the minor *trans* melting that overlaps with the exothermic reverse isomerization peak, the ΔH_{iso} for compound **2** could not be obtained. (d) DSC trace of uncharged PCM composite with 30% compound **1**, showing melting and crystallization of both TDA and *trans* Azo dopant.

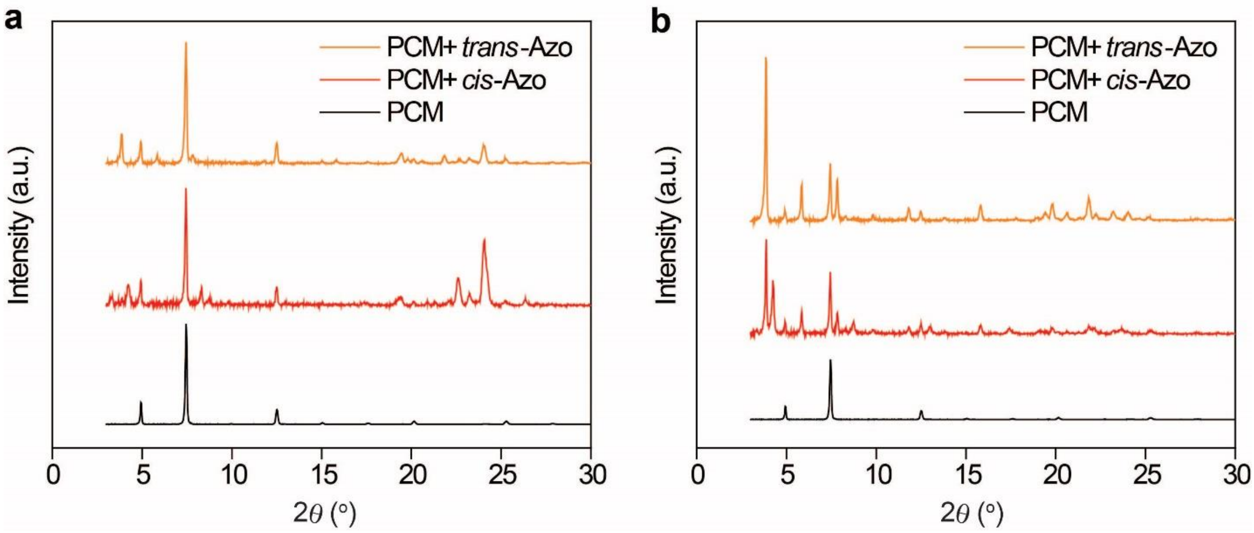

Supplementary Figure 4. PXRD patterns of PCM composites with (a) low doping (10%) and (b) high doping (45%) levels. We note that *trans*-Azo aggregation is significantly reduced at 10% doping level and that the PXRD patterns of uncharged and charged composites are similar. As predicted, the azobenzene dopants are sparsely distributed and ineffective in forming nucleating sites even in trans state, consistent with our DSC result (Supplementary Figure 2a) where the separate crystallization peak for *trans*-Azo is absent. In contrast, the PXRD of 45% doped composite shows that the charged PCM composite still possesses significant amount of *trans*-Azo aggregates, supporting our hypothesis on incomplete charging of dopants and the presence of *trans*-Azo aggregates (i.e. nucleators) leading to the reduced degree of supercooling.

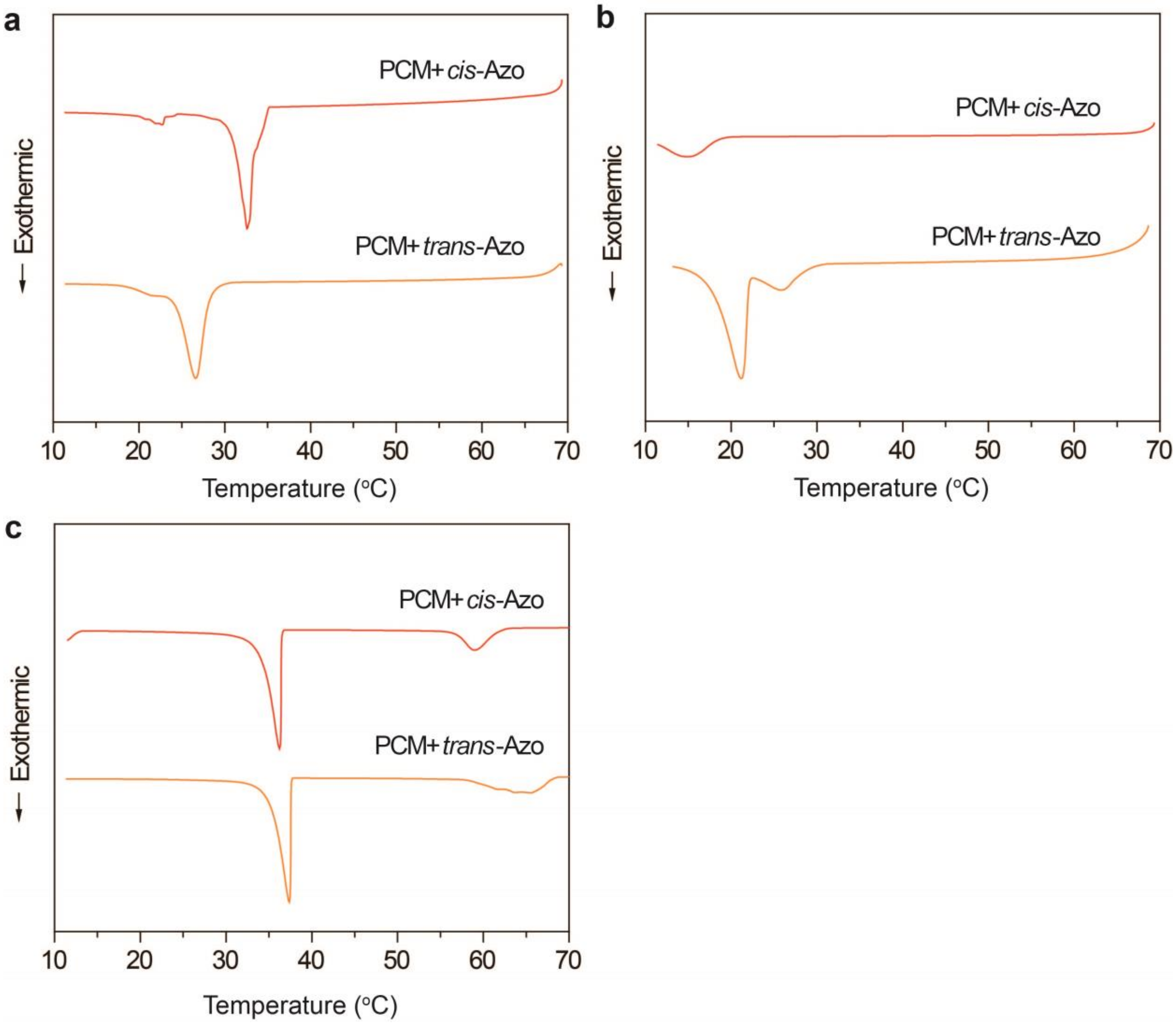

Supplementary Figure 5. DSC traces of composites with (a) 10 mol% *tbu*-Azo (compound **3**), (b) 40 mol% *tbu*-Azo (compound **3**), and (c) 30 mol% C_{10}-Azo (compound **2**) after and before solid-state charging. (a) Both the charged and uncharged composites crystallize congruently (the minor peak around 20 ^{o}C is from the crystallization of minor polymorph in PCM). (b) Uncharged composite shows the aggregation of *trans-tbu*-Azo and a separate crystallization of PCM. The charged composite shows a single peak assigned to the congruent crystallization of the composite. No crystallization of *cis-tbu*-Azo was found at temperatures down to −20 ^{o}C. (c) Uncharged composite shows two crystallization peaks, consistent with other composites with dopant **1** and **3**. The charged composite, however, is unique in that it shows both *trans*-C_{10}-Azo and *cis*-C_{10}-Azo crystallization (at 60 ^{o}C and 10 ^{o}C, respectively) due to the incomplete solid-state charging caused by strong dopant-dopant interactions (see Figure 4c).

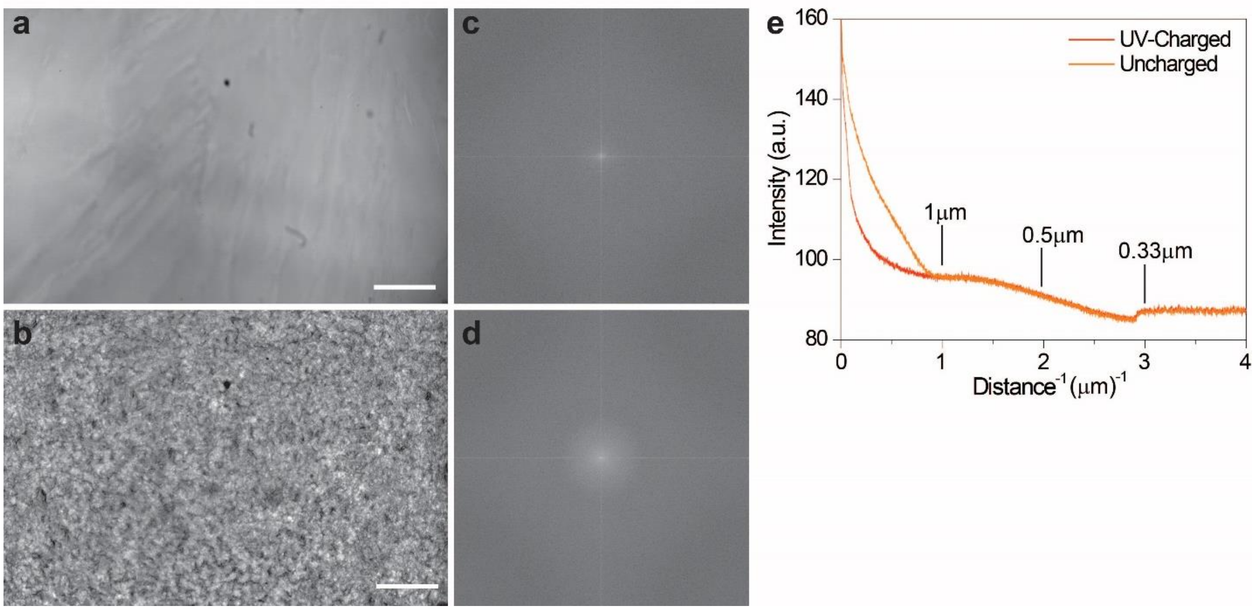

Supplementary Figure 6. Optical microscope images of composites containing 20 mol% *tbu*-Azo dopants (compound **3**) (a) after UV-charging for 1 h and (b) before charging, and their Fourier transforms (c, d). (e) Size distribution of the crystalline phase (aggregates) determined by the Fourier transforms. These images, taken at a temperature below 20 ^{o}C, exhibit different shapes of crystalline domains formed by the PCM. (a) Since the *cis* isomers of compound **3** remain in the liquid state upon cooling down to −20 ^{o}C, the PCM form large crystals, and the liquid azobenzene dopants are dispersed in the composite. (b) On the other hand, the *trans* isomers (T_c of 44 ^{o}C) crystallize congruently in the composite, forming smaller domains.

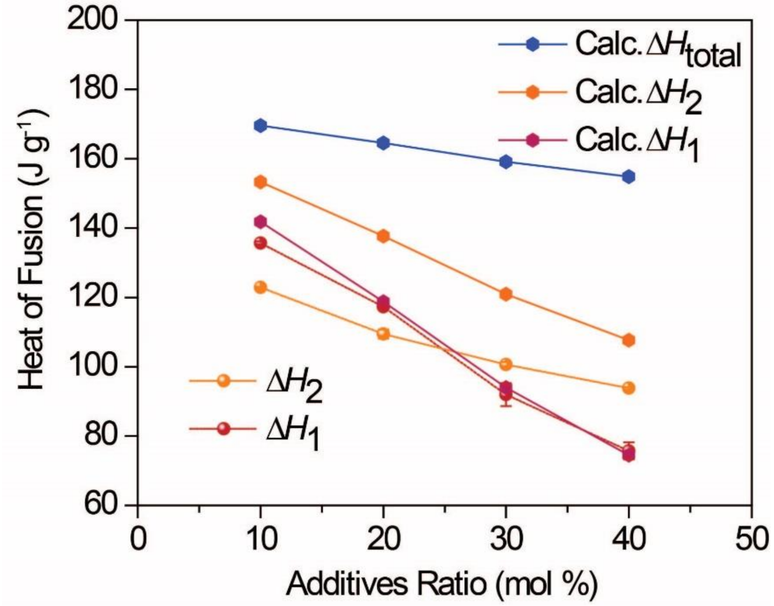

Supplementary Figure 7. Heat of fusion measured and calculated for composites containing compound **3** as dopants. Equations 1-3 in the main text are applied to the analysis of heat of fusion. $\Delta H_{cis\text{-Azo}}$=0 as *cis* compound **3** does not crystallize at T down to −20 ^{o}C. The measured and calculated ΔH_1 are very similar, while the measured ΔH_2 is considerably lower than the calculated values, implying more disruption caused by *trans* dopants than *cis*. The measured ΔH_1 and ΔH_2 follow the trend of T_1 and T_2 (thus the ΔT values). The measured exothermic ΔH of charged compound **3** during thermal reverse isomerization (ΔH_{exo}) is the sum of isomerization energy of azobenzene (ΔH_{iso}) and $\Delta H_{trans\text{-Azo}}$, since *cis* isomer is liquid and the *trans* is solid. ΔH_{exo} for compound **3** is 138.6 J g^{-1} which corresponds to 62 kJ mol^{-1}. ΔH decreases with higher doping levels marginally (87% of ΔH_{PCM} retained at 40% doping level). Error bars indicate standard deviations of data (heat of fusion) collected at least 5 times on each type of composite.

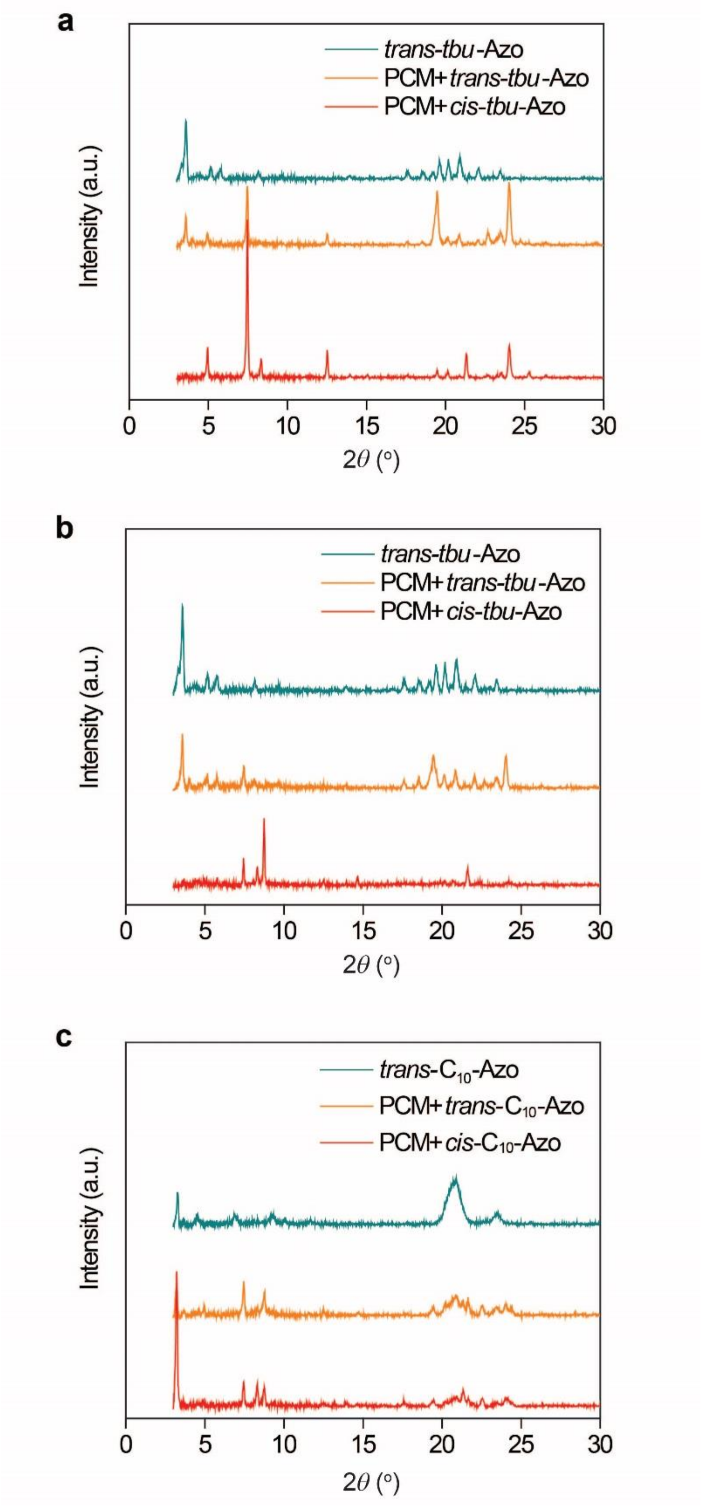

Supplementary Figure 8. Powder X-ray diffraction patterns of composites with (a) 10 mol% *tbu*-Azo (compound **3**), (b) 40 mol% *tbu*-Azo (compound **3**), and (c) 30 mol% C_{10}-Azo (compound **2**) before and after solid-state charging. The PXRD patterns of dopants (compound **2** and **3**) are shown for comparison with the composites.

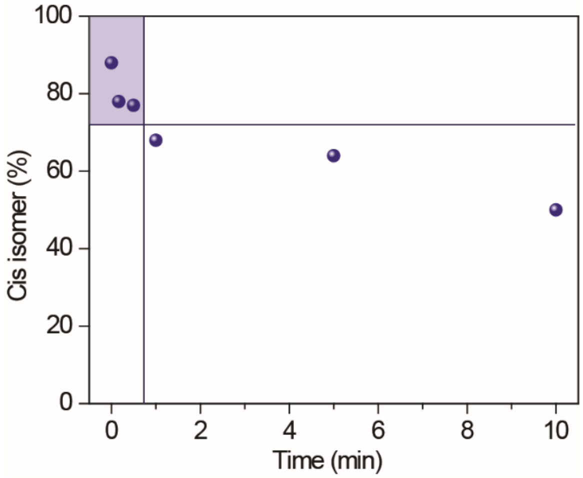

Supplementary Figure 9. Molar fraction of *cis* isomers measured by ^{1}H NMR during optical discharging (450 nm blue LED) of UV/thermally charged composites (30 mol% compound **1**). After 30 sec of blue light illumination, the composite crystallizes and the *cis* ratio drops below 70%. Half-life of the optical reversion under this condition is 10 min. The shaded area indicates the samples that conserve thermal energy in liquid state.

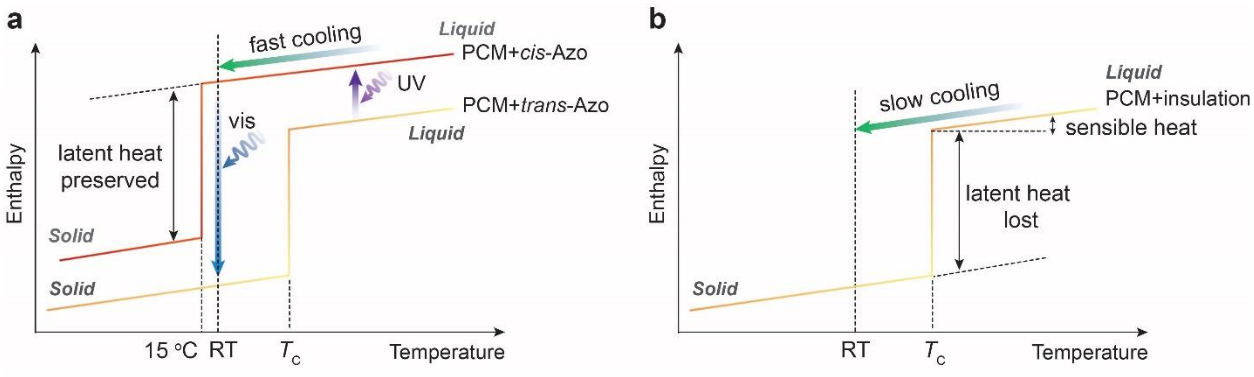

Supplementary Figure 10. Schematic energy diagrams of (a) azobenzene-doped PCM composite without thermal insulation and (b) conventional PCM with thermal insulation, during the cooling process from high temperature for thermal activation to room temperature. The primary role of thermal insulation is to decrease the cooling rate of the thermally activated materials and to reduce the loss of their sensible heat. Thermal insulation, however, can't stop the gradual heat transfer between the thermally charged PCMs and the cooler surroundings, eventually letting the reverse phase transition to occur and losing the latent heat when the temperature of PCMs drops below their crystallization temperatures. The PCM system with the photo-switching dopants, in the absence of thermal insulation, doesn't affect the cooling rate of the thermally activated PCMs. Instead, the dopants change the intrinsic property of PCMs, lowering the crystallization point down to room temperature and below; For example, the crystallization point of a UV-activated composite with 40% compound **3** is 15 °C. Without the insulation, the system may lose the sensible heat of PCMs fast, but the latent heat of PCMs is preserved at room temperature, which enables the transportation of the thermally activated PCMs retaining the large latent heat of known values. Also, another advantage of the system with dopants is the optically triggered heat release which doesn't require the change of the external temperature. In contrast, the PCMs stored in the insulated container have to be taken away from the insulating environment to be actively cooled down below the phase transition temperature. Otherwise, the insulated PCMs gradually lose the sensible and latent heat uncontrollably.

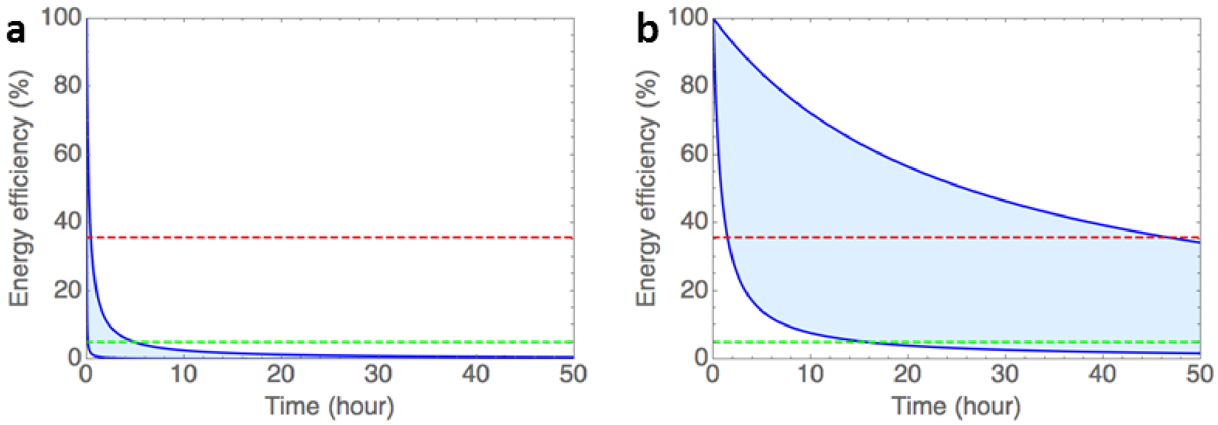

Supplementary Figure 11. Energy efficiency of pristine PCM (blue lines) with thermal insulation as a function of storage time. The blue area shows the range of variable results depending on the quality of insulation materials (thermal resistance values). Thickness of (a) 100 μm and (b) 1 cm for PCM and ΔT of 40 K were applied for the calculation, in the assumption of heating PCM to 60 ^{o}C and letting it spontaneously cool down to ambient temperature of 20 ^{o}C. The green dashed line represents the efficiency of current azobenzene-doped PCM system (100 μm thick) which needs initial (one-time charging) input of external photon energy (instead of continuous thermal energy input, as for conventional PCM) to maintain the liquid phase while storing thermal energy. The red dashed line suggests the improvement of energy efficiency in the system with another photo-switching dopant with 100% quantum efficiency.

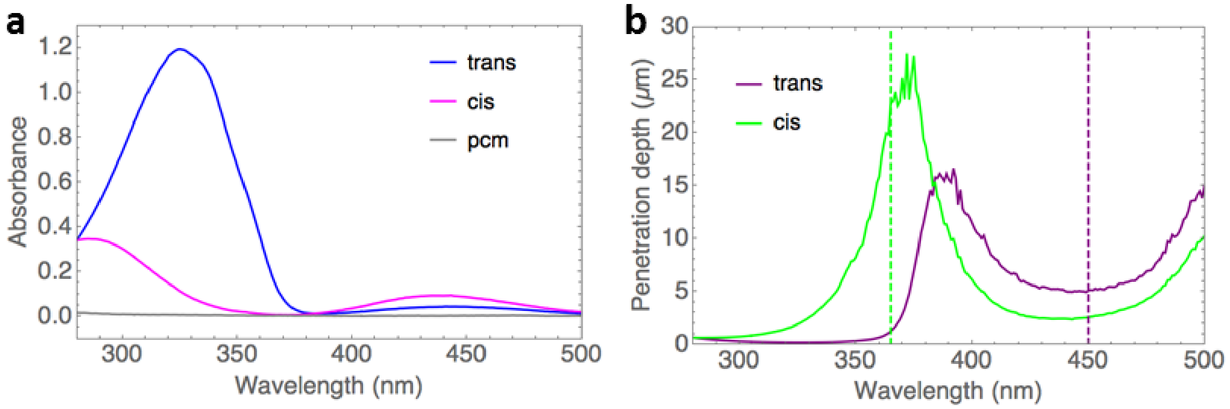

Supplementary Figure 12. (a) Absorbance of PCM, *trans*, and *cis* azobenzene in solution (ca. 10 μg mL^{-1}). (b) Estimated penetration depths of *trans* and *cis* azobenzene in thin film (35% doping), with the dashed lines indicating the UV (365 nm) and vis (450 nm) light that were used in the experiments for charging and discharging process. The intersections of dashed lines and the penetration profile give the penetration depth of the respective wavelength through composite films.

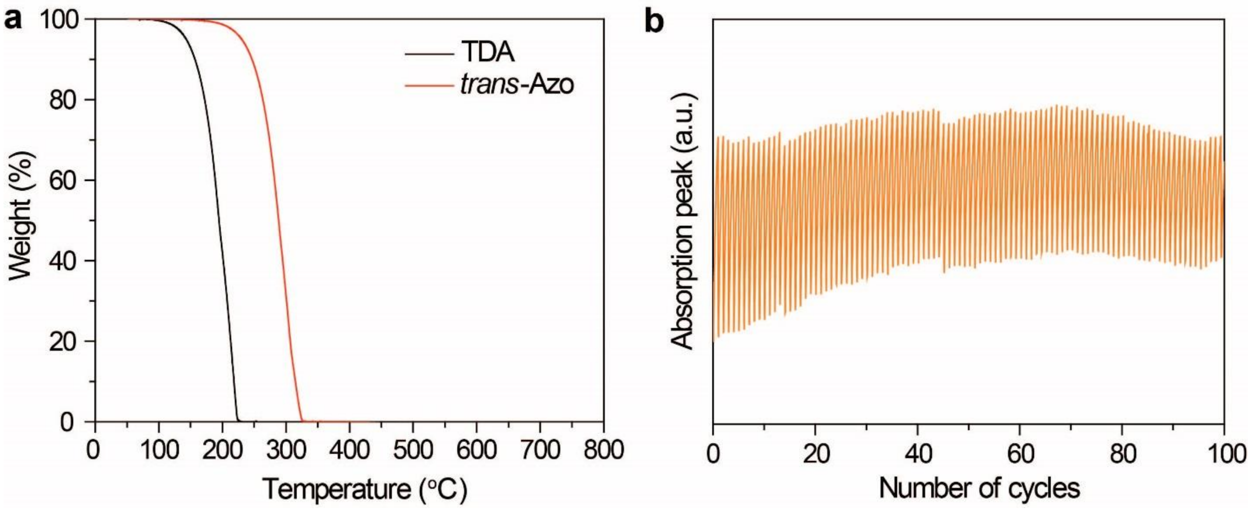

Supplementary Figure 13. (a) Thermophysical and (b) optical cycling stability of azobenzene dopant (compound **1**). (a) Thermogravimetric analysis (TGA) shows the high thermophysical stability of azobenzene dopant at temperatures up to 200 ºC. The thermal stability of the composite is rather determined by the lower decomposition temperature of the organic PCM. (b) Cycling was done by optical charging and discharging of azobenzene solution in dichloromethane using an arc lamp and filters to excite the π–π* and n–π* transitions of the *trans* and *cis* isomers, respectively. The absorption at 325 nm was recorded for over 100 cycles of charging and discharging for over 50 hours, demonstrating good stability of the azobenzene dopant towards repeated UV and visible light exposure (see Figure 2b in the main text for respective absorption spectrum of uncharged and charged azobenzene dopant).

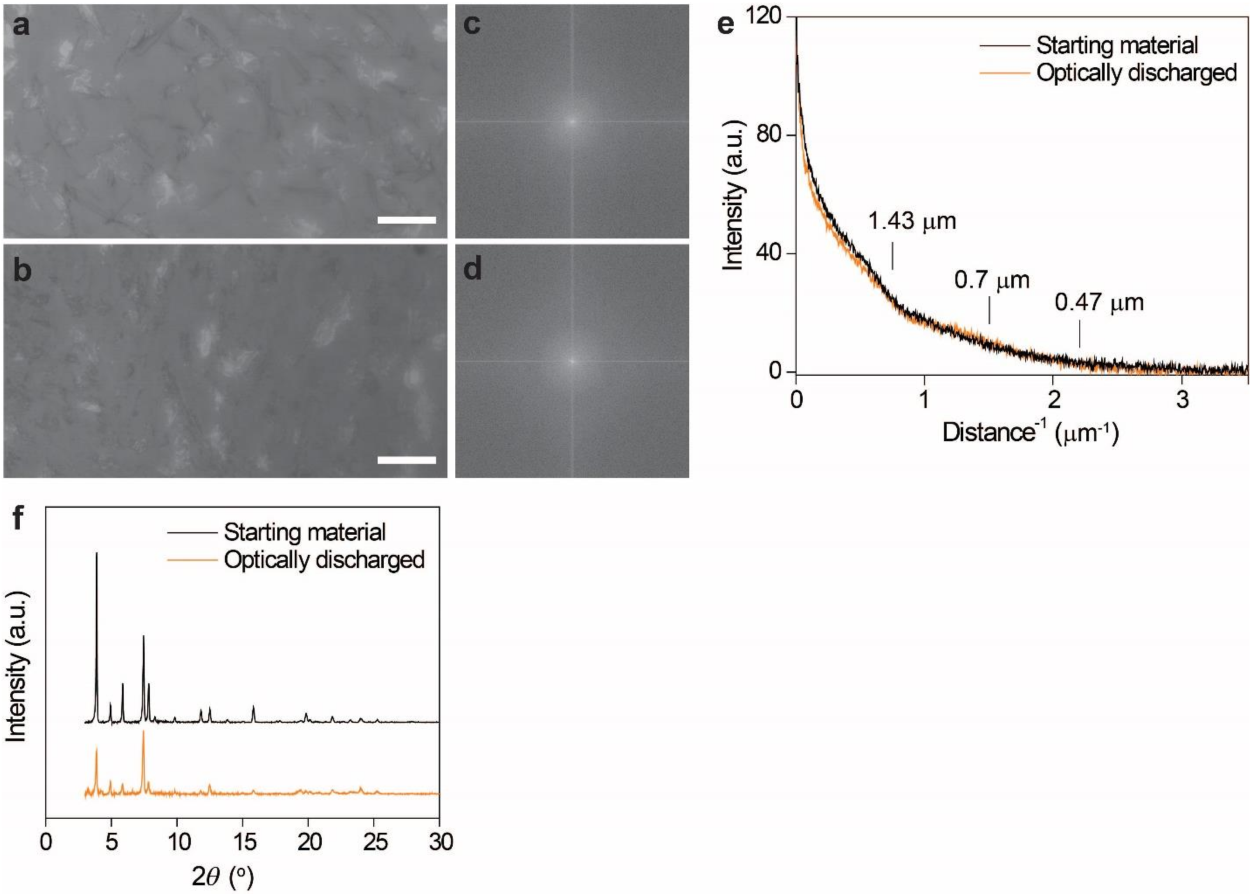

Supplementary Figure 14. Morphology and crystallinity of PCM composites before thermal activation (starting material) and after optically-triggered heat release. Optical microscope images of composites containing 30 mol% azobenzene dopants (compound **1**) (a) as prepared from solution and (b) after optical discharging, and their Fourier transforms (c, d). Scale bars are 50 µm. (e) Size distribution of the crystalline phase (aggregates) determined by the Fourier transforms. (f) PXRD patterns of the composites. The Fourier transform analysis of the images shows that size distributions of crystalline phase on two films are very similar, though the starting material possesses a slightly higher number of large crystallites than the optically discharged films. The PXRD patterns of the two samples are mostly identical in terms of the diffraction peak positions, while the relative intensity of the peaks assigned to PCM and to *trans*-Azo is different. In the optically discharged composite, the peaks representing *trans*-Azo aggregation are relatively suppressed. This may indicate that fast crystallization leads to less aggregation and incomplete *cis*-to-*trans* conversion, consistent with the molar fraction analysis of *cis* isomer during optical discharging (Supplementary Figure 9). Although the morphology of the PCM composite is slightly changed after the initial optical discharging, the morphology and crystallinity of the composite in the subsequent cycles of UV charging and visible light discharging will be fully reversible.

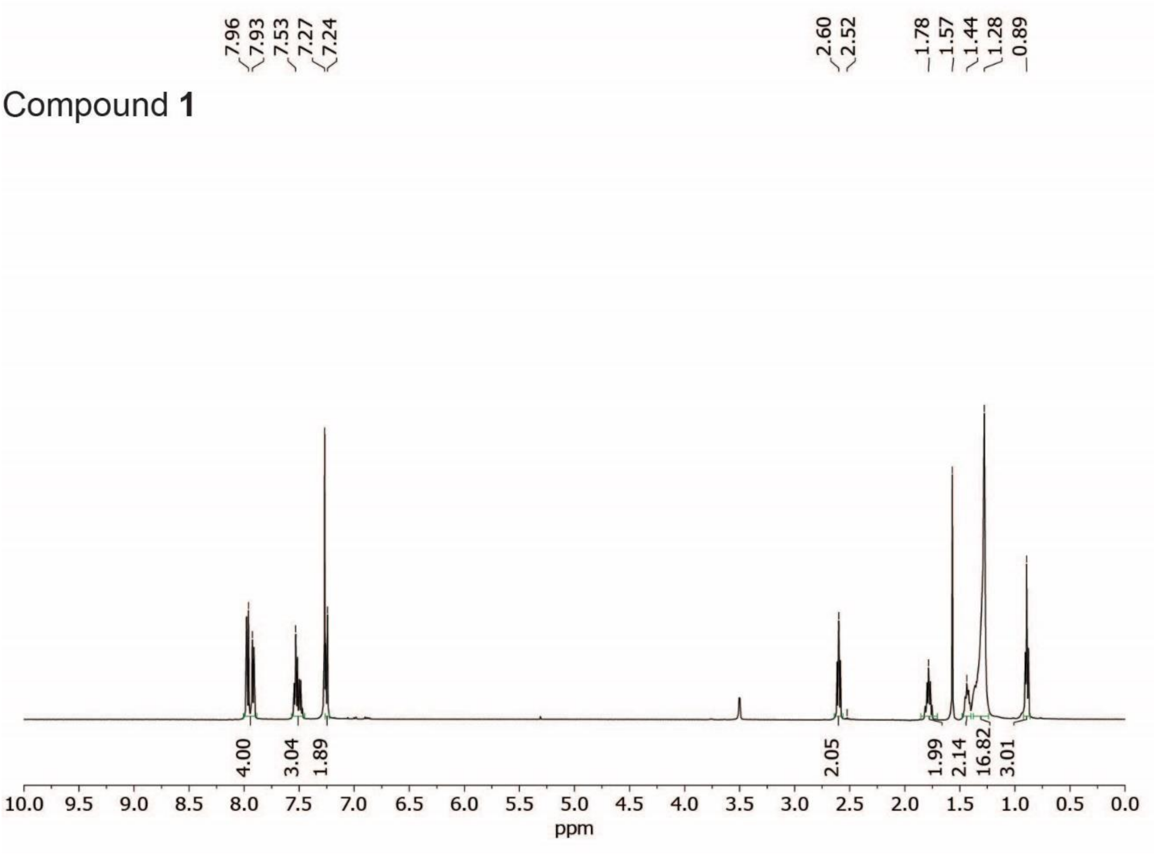

Supplementary Figure 15. ^{1}H NMR spectrum of compound **1.**

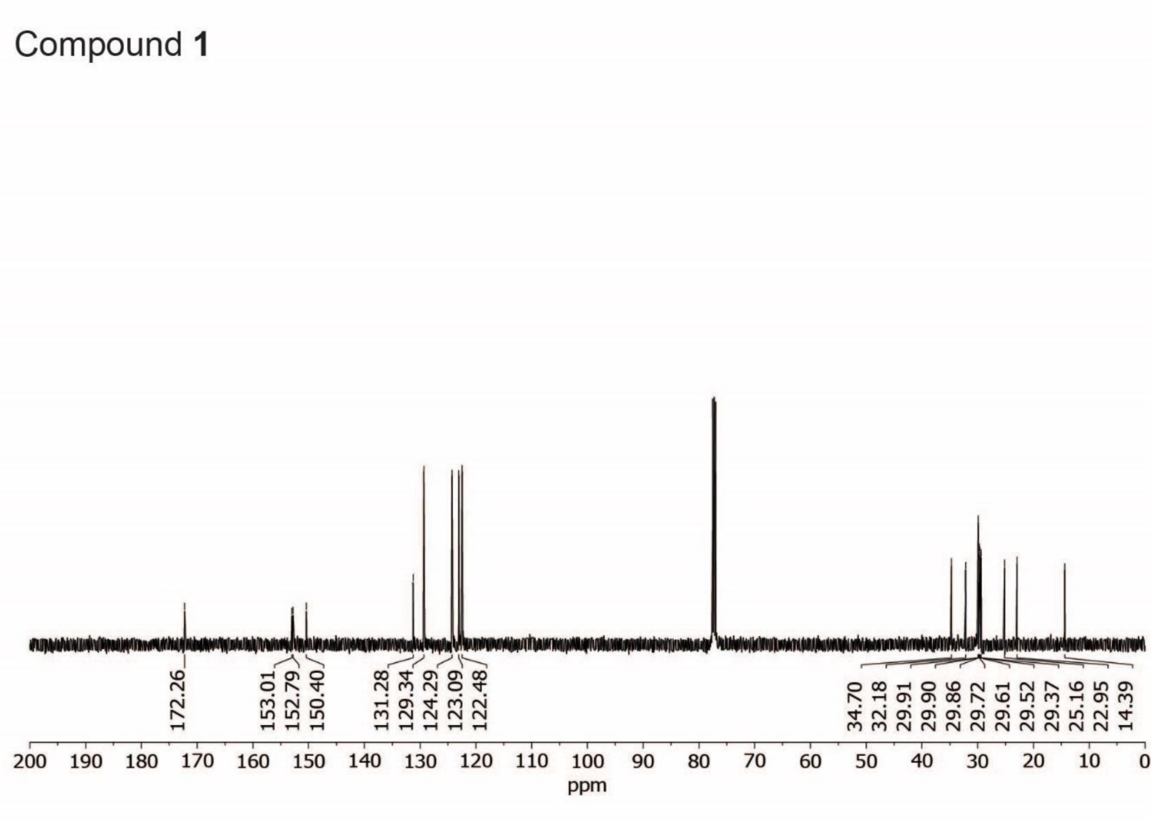

Supplementary Figure 16. ^{13}C NMR spectrum of compound **1**.

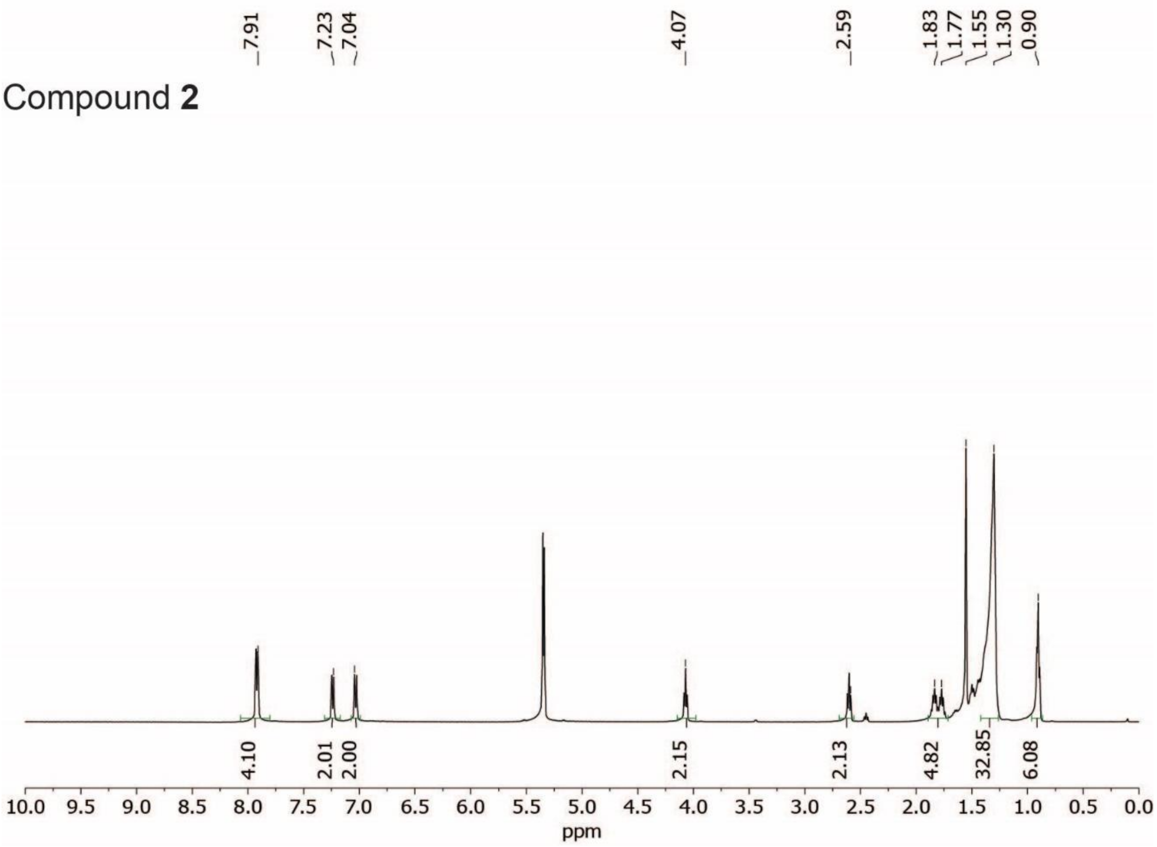

Supplementary Figure 17. ^{1}H NMR spectrum of compound **2.**

Compound **2**

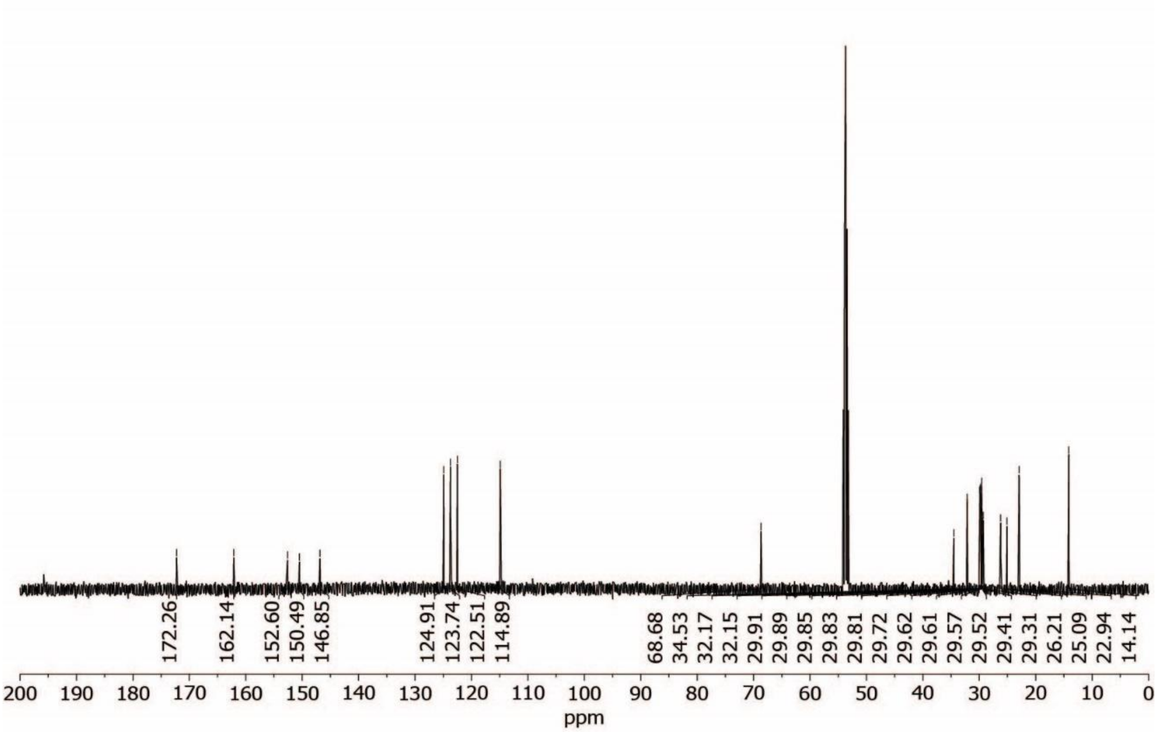

Supplementary Figure 18. ^{13}C NMR spectrum of compound **2**.

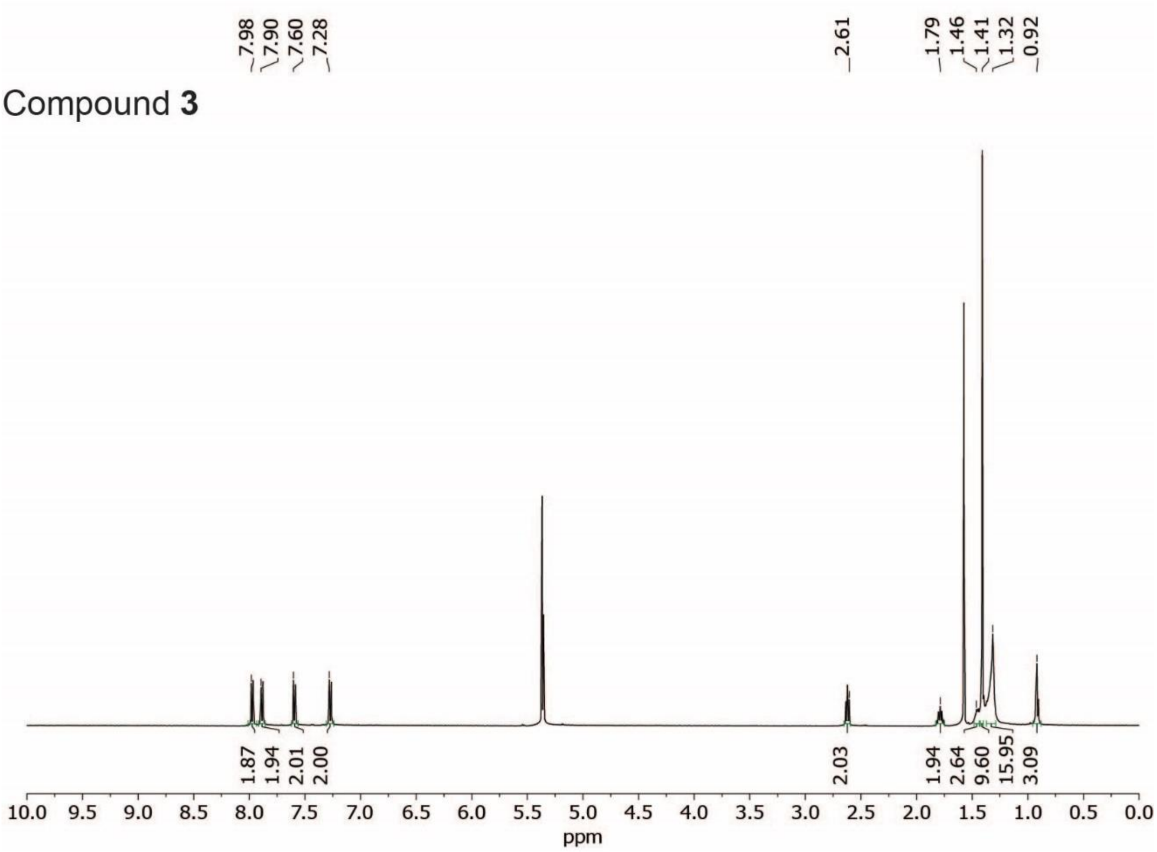

Supplementary Figure 19. ^{1}H NMR spectrum of compound **3.**

Compound **3**

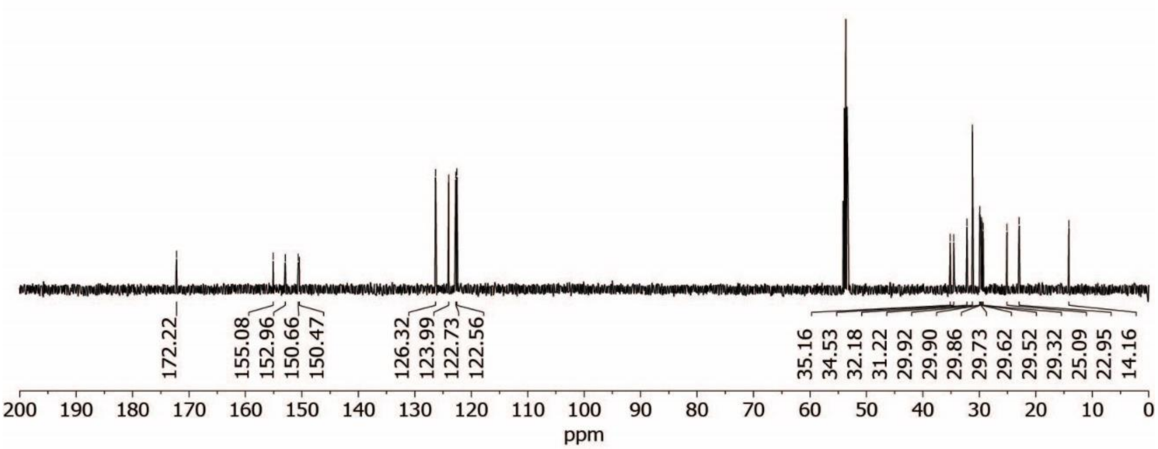

Supplementary Figure 20. ^{13}C NMR spectrum of compound **3**.

Supplementary Note 1. Discussion on the dispersion kinetics of azobenzene molecules in the composite.

For the class of PCM materials studied in this work, the rod-like geometry intensively restricts the end-over-end rotation and transverse motion. Therefore, the elementary diffusion processes in such highly entangled molecular systems are governed by longitudinal translations with segmental movements. This scenario has been demonstrated by previous measurements of density, self-diffusion coefficient, spin-lattice relaxation time, and X-ray diffraction on a variety of fatty acids.[1,2] While the activation energies can be affected by the local molecular structures, the diffusion coefficients in the temperature range relevant to our study (~300-315 K) are all at the order of 10^{-10} m^2 s^{-1}.In addition, the successful development of a linear model to predict the viscosity of multi-component fatty acid composite indicates that the diffusion mechanism of individual molecule in the mixture is similar to that in a single-phase material.[3]

Based on the above observations, we expect our system to exhibit a similar diffusion mechanism, although the exact diffusion coefficient *D* is difficult to predict because: 1) the diffusion unit of azobenzene is likely to be monomer rather than dimer resulting from the absence of strong hydrogen bonding between the COOH groups, which tends to increase *D* compared to the pristine PCM materials; 2) the enhancement of cross-section of azobenzene compared to PCM may suppress the diffusion processes; 3) the packing order may be affected by the dopants with additional π–π interactions, leading to further modification of *D*. Given a diffusion coefficient of 10^{-10}–10^{-9} m^2 s^{-1}, the time required for the dispersion of dopants in a sample domain with 1 mm radius (*r*) (as in Figure 2a) can be estimated as

$$t = \frac{r^2}{2D} = 500\text{–}\,5000\ \mathrm{s} \qquad (1)$$

This is consistent with our experiments that the dispersion had been completed within an hour. In the practical applications, however, the mechanical stirring will facilitate the mixing of the entire composite, so the diffusion process will have less impact on the charging time.

Supplementary Note 2. Qualitative analysis of the energy efficiency drop in pristine PCM thermal storage materials compared to the azobenzene-doped system.

Unlike the azobenzene-doped PCM composite, wherein the energy efficiency drop mainly arises from the inefficient switching of the current azobenzene dopants, the loss of efficiency in the pristine PCM without dopants is attributed to the energy flux required to maintain the temperature above the crystallization point by compensating for heat transfer to the environment. For pristine PCMs that are nonpolar homogeneous materials with no supercooling effect,[4,5] the energy efficiency should be 100% if the heat extraction occurs immediately after heat absorption. However, additional energy input is required to avoid spontaneous liquid-to-solid phase transition for longer storage, thus the efficiency decreases with increasing storage time. Using the range of thermal resistance (R value of 0.07–2.20 m^2 K W^{-1}) for common building insulation materials per 5 cm thickness,[6] we estimated the energy efficiency of pristine PCM based thermal storage system by

$$\eta = \frac{H_{\mathrm{PCM}}}{H_{\mathrm{PCM}} + \Delta T \times S \times t / R} \qquad (2)$$

where H_{PCM} is the heat storage by PCM, ΔT is the temperature difference between the heat storage material and surrounding environment, S is the surface area of the thin film, t is the thermal storage time, R is the ratio of the temperature difference across an insulator and the heat flux density. The result (Supplementary Figure 11) suggests that the energy efficiency of a traditional PCM device strongly depends on the quality of heat insulator, and can drop rapidly over time. In contrast, the energy efficiency of azobenzene-doped PCM system is constant over time in dark and determined by the initial charging efficiency. We note that the efficiency decay of a traditional PCM device is slower as the thickness increases, and we applied the thickness of 100 μm in this calculation in order to be consistent with the experimental condition that we used in the paper, as well as 1 cm in order to reflect the practical condition. Although the current energy efficiency of azobenzene-doped PCM system is 5–6% due to the low quantum yield of azobenzene photo-switching, the constant storage efficiency over a long period of time, without the need for continuous external energy input, can open up new opportunities particularly in the distribution of thermal energy storage materials.

Supplementary Note 3. Light penetration into the PCM composite and the propagating crystallization of PCM upon visible-light triggering.

Theoretically, the static penetration depths of *trans* and *cis* azobenzene are estimated by

$$\delta_{\mathrm{p},trans/cis} = \frac{d_{\mathrm{sol}} \times n_{\mathrm{sol}}}{A_{\mathrm{sol},trans/cis} \times n_{\mathrm{film}}} \qquad (3)$$

where $A_{\mathrm{sol},trans/cis}$ is the absorbance of *trans/cis* azobenzene in solution obtained by decomposing the absorbance spectra of initial and final states, d_{sol} is the thickness of the solution, $n_{\mathrm{sol/film}}$ is the dopant densities in solution / thin film. Within a static scenario where the dopants remain at the surface layer after the phase transition, the penetration of the UV and visible light are limited by the attenuation within the *cis* and *trans* azobenzene, respectively. The corresponding penetration depths are estimated to be 23 μm (for UV) and 5 μm (for vis) as illustrated in Supplementary Figure 12. The UV-vis absorption by PCM, i.e. tridecanoic acid, is extremely low within the range of wavelength in the study, but the penetration depths were calculated considering the impact of PCM matrix.

However, as we showed the complete charging of thicker powder samples (100–200 μm) and a large volume of azobenzene solution, a viscous or stirred liquid system can be charged far beyond the static penetration depth. Similarly, the 5 μm penetration depth of visible light through the discharged (i.e. *trans*) azobenzene will not limit the solidification process in such a stirring or flowing system. Even if the system is static, the initial formation of nucleating sites on the surface will enable the propagation of the crystallization throughout the whole PCM composite, and the heat release will not be limited by the visible light penetration depth.

According to homogeneous nucleation theory, the critical radius of nucleation (r*), i.e. minimum size of nucleus that can spontaneously grow, in a supercooled liquid needs to be first provided, and the molecular mobility of the supercooled liquid should be sufficient to allow the crystallization propagation.[7] Similar to the cited work describing an organic supercooled liquid with long alkyl functional groups that undergo propagation of crystallization throughout the entire area of stable liquid, once given crystalline seeds larger than r*, the propagation of the crystallization throughout our supercooled PCM composite below the original T_c can be initiated by the visible-light triggered formation of a nucleus. Then, given the significant mobility of the supercooled liquid containing sterically hindering *cis*-azobenzene dopants, the crystallization can propagate among the PCM molecules, phase-separating from the liquid *cis*-azobenzene dopants which has a low T_c of 9 ^{o}C. The energy gain by generating an energetically more favorable crystalline phase, exceeding the energy loss by the interface formation, is the driving force for the propagation. In our isothermal experiments at 36 ^{o}C, as shown in Figure 3c (main text), the crystallization propagates through static samples with various thicknesses (orders of magnitude greater than visible light penetration depth of 5 μm), supporting the theory. The crystallization of azobenzene dopants, however, is limited by the incomplete discharging of *cis*-azobenzene due to the low penetration depth, as seen in PXRD analysis (Supplementary Figure 14).

Supplementary Methods

Compound **1**. ^{1}H NMR (500 MHz, $CDCl_3$): δ 7.96 (d, 2H), 7.93 (d, 2H), 7.53 (m, 3H), 7.24 (d, 2H), 2.60 (t, 2H), 1.78 (m, 2H), 1.44 (m, 2H), 1.28 (m, 16H), 0.89 (t, 3H); ^{13}C NMR (500 MHz, $CDCl_3$): δ 172.26, 153.01, 152.79, 150.40, 131.28, 129.34, 124.29, 123.09, 122.48, 34.70, 32.18, 29.91, 29.90, 29.86, 29.72, 29.61, 29.52, 29.37, 25.16, 22.95, 14.39; HRMS (ESI; m/z): $[M + H]^+$ calcd. for $C_{25}H_{34}N_2O_2$, 395.2693; found, 395.2689.

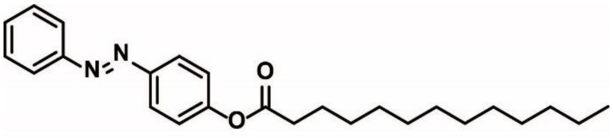

Compound **2**. ^{1}H NMR (500 MHz, CD_2Cl_2): δ 7.91 (dd, 4H), 7.23 (d, 2H), 7.04 (d, 2H), 4.07 (t, 2H), 2.13 (t, 2H), 1.83 (m, 4H), 1.30 (m, 32H), 0.90 (m, 6H); ^{13}C NMR (500 MHz, CD_2Cl_2): δ 172.26, 162.14, 152.60, 150.49, 146.85, 124.91, 123.74, 122.51, 114.89, 68.68, 34.53, 32.17, 32.15, 29.91, 29.89, 29.85(CH_2), 29.83, 29.81, 29.72, 29.62, 29.61, 29.57, 29.52, 29.41, 29.31, 26.21, 25.09, 22.94, 14.14; HRMS (ESI; m/z): $[M + H]^+$ calcd. for $C_{35}H_{54}N_2O_3$, 551.4207; found, 551.4220.

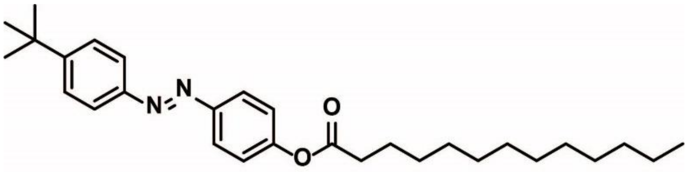

Compound **3**. ^{1}H NMR (500 MHz, CD_2Cl_2): δ 7.98 (d, 2H), 7.90 (d, 2H), 7.60 (d, 2H), 7.28 (d, 2H), 2.61 (t, 2H), 1.79 (m, 2H), 1.46 (m, 2H), 1.41 (s, 9H), 1.32 (m, 16H), 0.92 (t, 3H); ^{13}C NMR (500 MHz, CD_2Cl_2): δ 172.22, 155.08, 152.96, 150.66, 150.47, 126.32, 123.99, 122.73, 122.56, 35.16, 34.53, 32.18, 31.22, 29.92, 29.90, 29.86, 29.73, 29.62, 29.52, 29.32 25.09, 22.95, 14.16; HRMS (ESI; m/z): $[M + H]^+$ calcd. for $C_{29}H_{42}N_2O_2$, 451.3319; found, 451.3320.

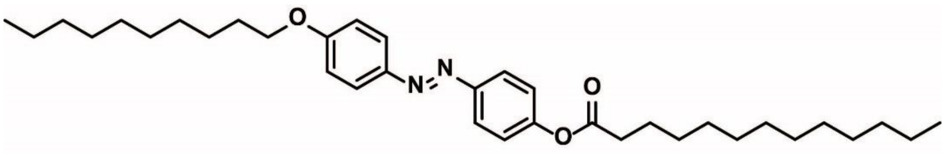

Supplementary References

1. Iwahashi, M. *et al.* Self-diffusion, dynamical molecular conformation, and liquid structures of n-saturated and unsaturated fatty acids. *J. Phys. Chem. B* **104**, 6186-6194 (2000).

2. Iwahashi, M., Yamaguchi, Y., Ogura, Y. & Suzuki, M. Dynamical structures of normal alkanes, alcohols, and fatty acids in the liquid state as determined by viscosity, self-diffusion coefficient, infrared spectra, and ^{13}C NMR spin-lattice relaxation time measurements. *Bull. Chem. Soc. Jpn.* **63**, 2154-2158 (1990).

3. Boyaci, I. H., Tekin, A., ÇIzmeci, M. & Javidipour, I. Viscosity estimation of vegetable oils based on their fatty acid composition. *J. Food Lipids* **9**, 175-183 (2002).

4. He, B. & Setterwall, F. Technical grade paraffin waxes as phase change materials for cool thermal storage and cool storage systems capital cost estimation. *Energ. Convers. Manage.* **43**, 1709-1723 (2002).

5. Baetens, R., Jelle, B. P. & Gustavsen, A. Phase change materials for building applications: A state-of-the-art review. *Energ. Buildings* **42**, 1361-1368 (2010).

6. Al-Homoud, D. M. S. Performance characteristics and practical applications of common building thermal insulation materials. *Build. Environ.* **40**, 353-366 (2005).

7. Chung, K. *et al.* Shear-triggered crystallization and light emission of a thermally stable organic supercooled liquid. *ACS Cent. Sci.* **1**, 94-102 (2015).
