# Supplementary material for: Optically-controlled long-term storage and release of thermal energy in phase-change materials
Source: Nat Commun. 2017 Nov 13;8:1446. doi: 10.1038/s41467-017-01608-y (PMC5684416; doi:10.1038/s41467-017-01608-y)
Supplement: Supplementary file 2 — Description of Additional Supplementary Files [file 41467_2017_1608_MOESM2_ESM.pdf]

Description of Additional Supplementary Files

File Name: Supplementary Movie 1
Description: Optical microscope movie of the UV-charged composite observed in bright and dark field to show the crystallization of the composite into smaller domains upon exposure to the focused white light. The width of movie is 550 μm.

File Name: Supplementary Movie 2
Description: Control movie of the uncharged composite. The width of movie is 550 μm.

File Name: Supplementary Movie 3
Description: Microscope movie of crystallization propagation within a charged PCM composite upon exposure to white and blue light. The diameter of liquid sample is about 4 mm. The PCM composite (30% compound **1**) was charged by UV irradiation and cooled to 36 ^{o}C on a thin glass substrate/DSC sensor under isothermal condition. The crystallization was recorded under white LED light by zipScope 2M USB Digital Handheld Microscope and accelerated by the exposure to 405-410 nm blue LED light. The movie plays 20 times faster than the real speed, and the crystallization of 5 mm domains generally take 5-10 min without constant blue light irradiation.
